# Mechanically robust stretchable semiconductor metallization for skin-inspired organic transistors

**DOI:** 10.1126/sciadv.ade2988

**Published:** 2022-12-21

**Authors:** Min Hyouk Kim, Min Woo Jeong, Jun Su Kim, Tae Uk Nam, Ngoc Thanh Phuong Vo, Lihua Jin, Tae Il Lee, Jin Young Oh

**Affiliations:** ^1^ Department of Chemical Engineering (Integrated Engineering Program), Kyung Hee University, Yongin, Gyeonggi 17104, Korea.; ^2^ Department of Mechanical and Aerospace Engineering, University of California, Los Angeles, Los Angeles, CA 90095, USA.; ^3^ Department of Materials Science and Engineering, Gachon University, Seong-nam, Gyeonggi 13120, Korea.

## Abstract

Despite recent remarkable advances in stretchable organic thin-film field-effect transistors (OTFTs), the development of stretchable metallization remains a challenge. Here, we report a highly stretchable and robust metallization on an elastomeric semiconductor film based on metal-elastic semiconductor intermixing. We found that vaporized silver (Ag) atom with higher diffusivity than other noble metals (Au and Cu) forms a continuous intermixing layer during thermal evaporation, enabling highly stretchable metallization. The Ag metallization maintains a high conductivity (>10^4^ S/cm) even under 100% strain and successfully preserves its conductivity without delamination even after 10,000 stretching cycles at 100% strain and several adhesive tape tests. Moreover, a native silver oxide layer formed on the intermixed Ag clusters facilitates efficient hole injection into the elastomeric semiconductor, which transcends previously reported stretchable source and drain electrodes for OTFTs.

## Teaser

A highly stretchable mechanically robust semiconductor meatllization is achieved through metal-polymer intermixing.

## INTRODUCTION

With rapid advances in bio-integrated electronics, devices are required to have a skin-like stretchable form, and there have been numerous studies to make electronic components stretchable (*1*, *2*). In electronics, a transistor is known as the most essential switching component; however, it is difficult to fabricate a transistor having mechanical stretchability. Organic thin-film field-effect transistors (OTFTs) have been considered as essential stretchable building blocks in the era of skin-inspired electronics (*3*–*5*). To obtain stretchable OTFTs for bioelectronics applications, all the materials they comprise, such as semiconductors, conductors, and dielectrics, must be mechanically stretchable with a high strain compliance and biocompatibility.

As intrinsically stretchable dielectrics, various commercialized elastomers, such as highly reliable cross-linked polydimethylsiloxane (PDMS), thermoplastic polyurethane, and styrene-ethylene-butylene-styrene (SEBS), have been successfully used; however, well-developed materials are still lacking to provide stretchability to semiconductors and conductors (*3*–*7*). Recently, massive efforts toward developing intrinsically stretchable semiconductors have led to many promising achievements (*3*–*12*). Unfortunately, only a few cases of stretchable electrodes for OTFTs have been reported but not fully studied, despite its importance (table S1).

In general, a key role of the electrode in the stretchable OTFT is to inject electric current into the semiconductor, which requires appropriate work function, mechanical stretchability, and durability. As a stretchable electrode for the stretchable OTFTs, single-wall carbon nanotubes (SWCNTs) having a high work function and moderate electric resistance have been widely used because electrical percolation in their network structure is well maintained under mechanical strain (*13*–*16*). However, in terms of biocompatibility, SWCNTs are toxic and can cause inflammatory responses, malignant transformation, DNA damage, and mutation, all of which are still under debate in the research field of biomaterials (*17*–*19*). Consequently, the use of SWCNTs in bioelectronics has been declining gradually (*20*). Poly(3,4-ethylenedioxythiophene):poly(styrenesulfonate) (PEDOT:PSS) has been considered as another material for stretchable electrodes. It has intrinsic flexibility with a high work function and is able to form a smooth electrical contact with other organic semiconductors (*21*–*23*). Methods of incorporating stretchability in PEDOT:PSS electrodes include either blending plasticizers with PEDOT:PSS or a newly designed cross-linkable PEDOT:PSS with elastic polymers (*21*, *23*). Although PEDOT:PSS has an attractive merit in terms of excellent hole injection for OTFTs, its inherent air instability due to oxidation and relatively low electrical conductivity may limit its use in future bioelectronic applications (*24*–*27*).

Noble metal deposition through various methods such as physical vapor deposition, ion implantation, and chemical reduction has been suggested for stretchable and soft electrodes (*28*–*32*). Recently, microcrack-based noble metal films on insulating elastomers such as PDMS and SEBS have been reported as an alternative stretchable electrode for organic electrochemical transistors and OTFTs (*8*, **33**). The microcracked Au film on elastomers maintains their conducting pathway while releasing applied stress, which makes it a stretchable electrode; however, there are problems such as continuous change in electrical resistance and microscale morphological instability on strain. Moreover, a serious drawback of the microcrack-based stretchable electrodes is delamination wear under repeatable mechanical deformation due to its poor adhesion. Adhesion strength in withstanding all-round deformation needs to be considered as another important requirement for stretchable electrodes because hard packaging is not available in bioelectronics, as the sensation of foreign substances in contact with human skin should be minimized. The aforementioned studies have not taken into consideration the delamination wear, and their technology readiness level has not yet reached the stage where mechanical durability has been considered for stretchable semiconductor devices.

In this work, we introduce a mechanically robust stretchable semiconductor metallization based on silver (Ag) metal-elastic semiconductor intermixing that can meet all the requirements for the stretchable OTFTs. As a noble metal, Ag has the highest electrical conductivity among metals, has a reasonable price compared to Au, and has easier processability than SWCNT, which is conventionally used in organic electronics. The metal-elastic semiconductor intermixing occurred during thermal evaporation, allowing silver atoms to diffuse into the elastomeric semiconducting film, which can be systematically controlled by the metal deposition rate, enabling mechanically durable and highly stretchable semiconductor metallization. The stretchable Ag metallization exhibited a high tolerance to more than 10,000 stretches at 100% strain without mechanical delamination, while it injected the charge carriers efficiently into the elastic semiconductor without changes in resistance even under 100% strain. Using the highly stretchable, electrically conductive, and even mechanically durable Ag metallization of intrinsically stretchable semiconductor surfaces, skin-like stretchable OTFTs were successfully fabricated. Last, we demonstrated a fully stretchable strain-insensitive 5 × 5 active-matrix OTFT array for skin-inspired electronics.

## RESULTS

**Fig. 1.**
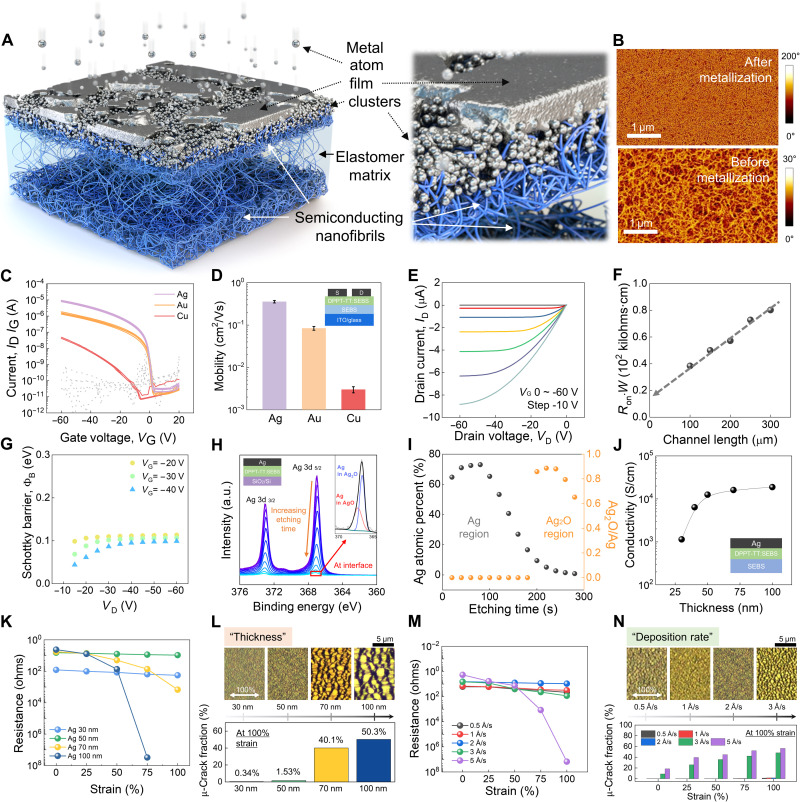
Stretchable metallization and characterizations. (**A**) Schematic of stretchable metallization. (**B**) AFM images of surface morphology before and after metallization on a nanofiber-based semiconducting film. Scale bars, 1 μm. (**C**) Transfer curves and (**D**) field-effect mobilities of organic field-effect transistors (OFETs) with different metal electrodes (Ag, Au, and Cu). (**E**) Output curve of OFET with a Ag-metallized electrode. (**F**) Resistance of OFET with Ag electrode as a function of channel length for extracting contact resistance. (**G**) Calculated Schottky barrier of OFET with Ag electrode as a function of drain voltage and gate voltage. (**H**) XPS spectra in the range of 360 to 376 eV, showing Ag 3d peak as a function of etching time (inset: XPS of Ag 3d 5/2 peaks containing silver oxide, AgO, and Ag_2_O information). a.u., arbitrary units. (**I**) Ag atomic percent and Ag_2_O/Ag ratio of the Ag-metallized film as a function of etching time. (**J**) Conductivity of the Ag-metallized film as a function of thickness on elastic substrate. (**K**) Resistance of the Ag film on an elastic substrate with different thicknesses as a function of strain. (**L**) Optical microscope (OM) images of different thicknesses of Ag-metallized film after 100% stretching (top) and crack fraction on that strain % (bottom). (**M**) Resistance of Ag films with various deposition rate conditions on strain. (**N**) OM images of different deposition rates of Ag-metallized film after 100% stretching (top) and crack fraction on that strain % (bottom).

Figure 1A illustrates Ag metallization of an elastic semiconductor. When Ag is deposited on the elastic semiconductor using a high-vacuum thermal evaporator, some Ag atoms with high kinetic energy diffuse and start to form Ag nanoclusters inside the surface of the elastic semiconductor layer, and the others with low energy start to nucleate and grow on the surface. As interconnecting the Ag nanoclusters inside and Ag thin film on the surface, the Ag metallization is completed in the form of a continuous thin film rooted in the elastomeric semiconductor, where there is a Ag cluster–elastic semiconductor intermixing region that acts as a physical glue layer, which provides the Ag film with high mechanical stretchability and strong adhesion. Formation of the continuous thin film was observed from atomic force microscopy (AFM) phase images of the nanofiber-based elastic semiconductor film before (bottom) and after (top) metallization (Fig. 1B).

To verify the capability of Ag metallization as source/drain electrodes, we fabricated OTFTs with a thin-film elastic semiconductor consisting of a SEBS elastomer and nanofiber-based poly(2,5-bis(2-octyldodecyl)-3,6-di(thiophen-2-yl) diketopyrrolo[3,4-c] pyrrole-1,4-dione-alt-thieno[3,2-b] thiophen) (DPPT-TT) through the nanoconfinement effect (fig. S1) (*4*, *11*). The DPPT-TT nanofibrils were observed in the AFM images (Fig. 1B). The OTFTs showed higher mobility (0.36 cm^2^/Vs) with Ag than the other noble metals (Cu: 0.003 cm^2^/Vs and even Au: 0.084 cm^2^/Vs) (Fig. 1, C and D), although Au is expected to be the most suitable p-type ohmic contact for OTFTs owing to its high work function (5.0 eV) compared with the other noble metals (Cu: 4.7 eV and Ag: 4.3 eV) and lower barrier (Φ_B_) for hole injection into polymer semiconductors (**34*–*36**) . Moreover, the OTFTs with Ag metallization exhibited typical ohmic-contact output curves (Fig. 1E). Ag exhibited the lowest contact resistance (*R*_C_) (0.17 megohms) among the metals (Au and Cu), which is comparable to that of previously reported stretchable CNT and PEDOT:PSS electrodes (Fig. 1F and figs. S2 to S4) (*4*, *23*), and its effective hole injection barrier was approximately 0.1 eV, which is negligible compared to the conventional injection barrier of more than 0.2 eV (Fig. 1G and fig. S5) (**37**).

To elucidate the reason for these results, the work function of the Ag electrode was measured using photoelectron spectroscopy in air (fig. S6) as 4.77 eV, which is higher than the theoretical value (4.3 eV) in vacuum. According to the literature, the work function (Φ_W_) of Ag is known to be in the range of 4.3 to 4.8 eV, depending on the environment or processing (**37**, **38**). It is thought that the Ag metallization in this work has a sufficiently high work function to form an ohmic contact. To gain further insight into the superior hole injection, the composition change at the interface between Ag and the polymer semiconductor in the depth direction was analyzed using x-ray photoelectron spectroscopy (XPS) depth profiling, and the results showed that a native silver oxide layer (Ag*_x_*O) having a high work function (Φ_W_ = 5.2 eV) is formed at the interface, as shown in Fig. 1 (H and I) and figs. S7 and S8, which implies that the native oxide could be superior to Au (Φ_W_ = 4.6 eV, measured in air; fig. S6B) in creating a low-resistance ohmic contact for hole injection into the elastic semiconducting film (highest occupied molecular orbital level = 5.04 eV; fig. S6C). The presence of Ag*_x_*O could result in the Ag electrode having the lowest contact resistance (*R*_C_) and an effective injection barrier (Φ_B_).

Electrical conductivity of the Ag metallization as a function of thickness defined by a thickness monitor was estimated as shown in Fig. 1J. As the thickness increased, electrical conductivity increased sharply to 12,532 ± 409 S/cm (50 nm) and then leveled off (metallic conductivity, >10^4^ S/cm). This result reflects the formation of a continuous Ag thin film formed after approximately 50 nm of deposition. The similar thickness dependence of electrical conductivity was observed in Ag metallization even on a SEBS elastomer substrate (fig. S9). Moreover, the initial resistance of the Ag metallization on the elastomeric semiconducting film was maintained over half a year (193 days) in air at the same level as those stored in a glove box (fig. S10). The mechanical stretchability of the Ag metallization was substantially affected above and below an approximate value of 50 nm. Ag electrodes of thickness less than 50 nm almost preserved their initial resistance even at 100% strain without electrical sacrifice, as shown in Fig. 1K, which presents the highest conductivity or lowest resistance among previously reported stretchable electrodes for the stretchable organic transistors (table S1). The microscale morphology of the 50-nm Ag metallization on an elastic semiconductor under pristine (0% strain) and 100% strain conditions was investigated using an optical microscope (OM), as shown in fig. S11, and hardly any microcracks could be observed in the two OM images. In contrast to the case of Ag, several microcracks were observed in the other noble metals (Au and Cu) over all thickness range from 30 to 100 nm under 100% strain (figs. S12 and S13). The fraction of crack area for all noble metal films was quantified as shown in figs. S14 to S17. While Au and Cu films showed several microcracks from 25% strain for all thickness ranges (30 to 100 nm) with highly strain-sensitive resistance change resulting in electrical disconnection (fig. S18), in the case of Ag, only several microscale cracks were observed for samples with thickness of greater than 50 nm at 100% strain (Fig. 1L). There was an optimal thickness (50 nm) of Ag metallization that consisted of the metal-elastic semiconductor intermixing region and pure metal region to balance the electrical conductivity and mechanical stretchability.

In addition, we found that the deposition rate of Ag is a significant processing parameter to affect the crack morphology and resistance change of metallization under strain (Fig. 1, M and N, and figs. S19 and S20). A faster rate of deposition of more than 3 Å/s makes the metallization strain sensitive with a large crack area fraction (>48%), whereas the metallization has a relatively strain-insensitive resistivity with negligible crack area fraction below 2 and up to 100% strain at a slower rate of deposition (0.5 to 1 Å/s). This result reflects that the formation of metal-elastic semiconductor intermixing can be affected by the deposition rate. A faster deposition rate leads to a faster metal film formation on the surface because newly deposited Ag atoms are consumed in the growth of the thin film rather than diffusing inside. A slower deposition rate of Ag metallization provides a higher probability of forming a metal-elastic semiconductor intermixing region, yielding higher initial resistance and a gradual increase in resistance on strain due to the thinner metal layer. Thus, a moderate deposition rate (2 Å/s) for metallization was applied to mechanical robust stretchable metallization, balancing between metal-elastic semiconductor intermixing for adhesion and metal layer for resistivity. In contrast, other noble metals have no correlation between the deposition rate of metals and crack formation under strain. For all deposition rates, Au and Cu films showed strain-sensitive resistance and severe microcrack formation on strain (figs. S21 to S25).

**Fig. 2.**
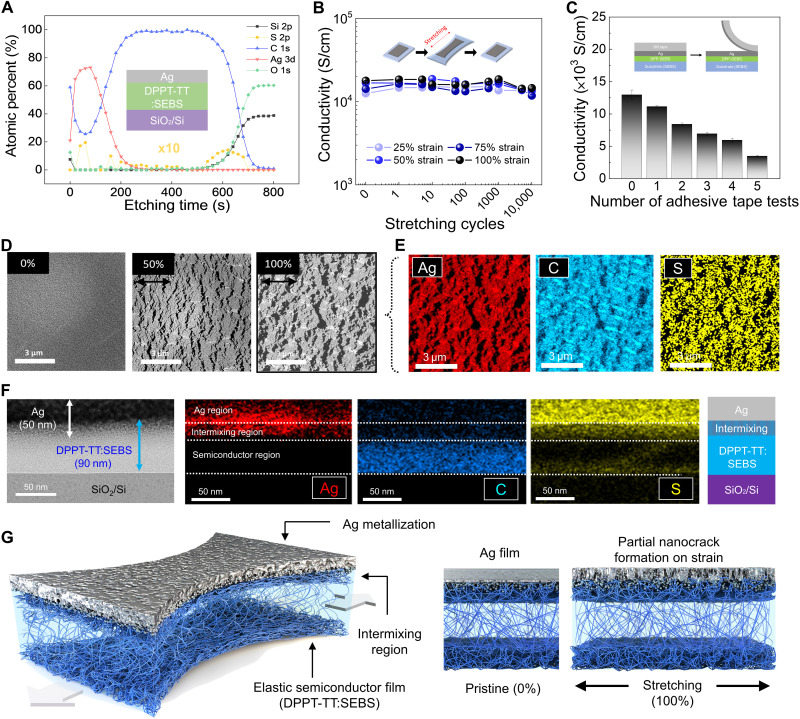
Mechanical robustness of stretchable metallization and mechanism analysis. (**A**) XPS depth profiling of the Ag-metallized film (inset: sample structure for XPS analysis). (**B**) Conductivity changes of the Ag-metallized film on an elastic substrate at different strains (25, 50, and 100%) during multiple stretches up to 10,000 cycles. (**C**) Conductivity changes of the Ag-metallized film on an elastic substrate during adhesive tape tests. (**D**) STEM images of the Ag-metallized film on an elastic substrate with 0, 50, and 100% strain and (**E**) EDS component mapping images (Ag, C, and S atoms) of the Ag-metallized film at 100% strain. Scale bars, 3 μm. (**F**) Cross-sectional TEM images and EDS component mapping images for Ag, C, and S atoms. Scale bars, 50 nm. (**G**) Schematic of the suggested mechanism of mechanically robust stretchable metallization on an elastic semiconducting film.

To obtain direct evidence of the existence of the metal-elastic semiconductor intermixing, XPS depth profile analysis of metallization films was performed as shown in Fig. 2A and fig. S26. A metal-elastic semiconductor intermixing region (atomic percent in the middle of metal region: Ag: 73.1% and C_Ag_: 26.9%, indicating DPPT-TT:SEBS composite) was observed in the Ag metallization, whereas other noble metals have pure metal atomic percent in metal region (Au: 95%; Cu: 99%) and elastic semiconducting film (C_Au_: 5%; C_Cu_: 1%), which indicates that there is no metal-elastic semiconductor intermixing. Compared to other noble metals, Ag is known to have a high diffusion coefficient in polymers. The relative surface diffusion coefficients for Ag, Au, and Cu are 100, 2, and 1, respectively, at room temperature (**39**). Thus, when Ag, with a superior diffusion rate, is deposited on an elastic polymer semiconductor film, a mixing layer could be formed, and it is possible for the film to have a strong mechanical adhesion.

To evaluate the stretching endurance of the Ag-metallized electrode (50 nm), its electrical resistance was tracked during 10,000 stretches with 25, 50, 75, and 100% strain amplitudes, respectively (Fig. 2B). The Ag-metallized electrode almost preserved its initial electrical resistance without plastic deformation even after severe multiple stretches (fig. S27). Furthermore, to estimate the adhesion durability of the Ag-metallized electrode under harsh conditions, a delamination wear test was performed using commercial adhesive 3M tape (3M 810 tape, adhesion force: 2.2 N/cm; fig. S28 and movie S1), and the results thus obtained are presented in Fig. 2C. The initial morphology and electrical conductivity of the Ag-metallized electrode were unexpectedly almost preserved without delamination, even after five tape detaches (fig. S29). Because a noble metal forms a physical binding to an organic polymer instead of a chemical bonding, it is known that a deposited noble metal film is easily delaminated from an elastic organic polymer even by a very weak external force (**40**, **41**). However, the Ag metallization in this study exhibited unprecedented stretching endurance and adhesion durability.

To further investigate the strong adhesion of the deposited Ag film on the elastic semiconducting polymer, the nanoscale morphology of the Ag film under strain was determined using scanning transmission electron microscopy (STEM) and AFM (fig. S30). Figure 2D presents the STEM images of the 50-nm-thick Ag film under various strains (0, 50, and 100%). The pristine Ag film (0% strain) exhibited a uniform surface morphology. When the strain increased to 50%, the film started to tear and exhibited nanoscale cracks instead of delamination as a method of releasing the external stress. When the strain further increased to 100%, the cracks enlarged more but did not catastrophically propagate through the film, maintaining the geometrical connectivity of the Ag film. Element mapping results from the energy-dispersive x-ray spectroscopy (EDS) of the STEM image under 100% strain show that Ag, sulfur (S) of DPPT-TT, and carbon (C) of DPPT-TT have almost the same spatial distribution with those without stretching (Fig. 2, D and E, and figs. S31 to S33). This result implies that Ag and the semiconductor polymer are strongly bound to each other as they are not mutually spaced apart under strain.

Furthermore, to reveal the reason for the strong adhesion, the cross section of the interface between the 50-nm Ag film and elastic semiconductor polymer was directly observed through TEM (fig. S34). Figure 2F presents a cross-sectional TEM image and the EDS mapping at the interface. The results indicate that there are three separate layers (top: 20-nm-thick Ag; middle: 30-nm-think Ag-semiconductor mixture; and bottom: 60-nm-thick elastic semiconductor polymer). Ag atoms and polymers could have an extremely large number of physical binding sites in the intermixing layer, forming strong adhesion. Besides the contribution to adhesion, such an intermixing Ag-polymer structure also significantly contributes to the high stretchability and conductivity of the Ag metallization. The comprehensive mechanism of the highly stretchable and adhesive electrode on elastic semiconductor is schematically illustrated in Fig. 2G.

**Fig. 3.**
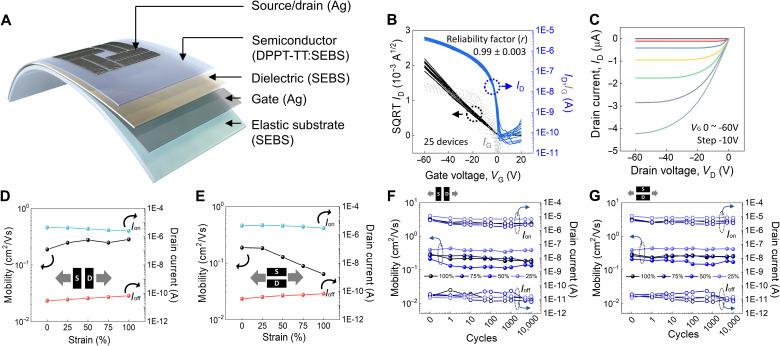
Fully stretchable OFET characterization. (**A**) Schematic of fully stretchable OFET structure. (**B**) Transfer curves of all devices and (**C**) typical output curve of a fully stretchable organic transistor. (**D**) Mobility and on/off current ratio of a fully stretchable organic transistor as a function of parallel stretching directions and (**E**) perpendicular directions to channel current direction. (**F**) Field-effect mobility and on/off current ratio changes of fully stretchable transistors at different strains (25, 50, 75, and 100%) during multiple stretching cycles up to 10,000 times for parallel and (**G**) perpendicular stretching directions.

With these advantages of stretchable Ag metallization, we fabricated fully stretchable OTFTs in a top-contact-and-bottom-gate structure with Ag electrodes, DPPT-TT/SEBS nanoconfined stretchable semiconductor, and a SEBS dielectric, as shown in Fig. 3A and fig. S35. The devices exhibited typical transfer curves and output performance, as shown in Fig. 3 (B and C). The average field-effect mobility of the 25 devices was 0.288 cm^2^/Vs with a reliability factor of 0.99 ± 0.003, which indicates that the fully stretchable devices are highly reliable in terms of charge carrier transport (**42**, **43**). Moreover, the transistors have successfully worked at low operating voltage down to 5 V with a similar shape of transfer curve (fig. S36). However, for further higher on-current at low operating voltage, the current devices require high-mobility semiconductor and high-*k* dielectric materials. When the device was stretched along the channel length and width direction of up to 100% strain, the devices maintained their field-effect mobilities and on-off ratios near the values of 0.183 cm^2^/Vs and 3.94 × 10^4^ as shown in Fig. 3 (D and E), respectively (fig. S37). Even under a 100% cyclic tensile strain applied more than 10,000 times, the fully stretchable OTFTs maintained their initial electrical properties irrespective of the stretching direction (Fig. 3, F and G, and figs. S38 to S45). To the best of our knowledge, this is the harshest stretching durability test among those previously reported for highly reliable stretchable OTFTs. Moreover, the stretchable OTFT was successfully operated on even biaxial strain up to 30% while maintaining the initial electrical performance (fig. S46). This mechanically robust and stretchable Ag metallization was also verified on another promising semiconducting polymer (indacenodithiophene-*co*-benzothiadiazole):SEBS blend system as shown in figs. S47 to S49.

**Fig. 4.**
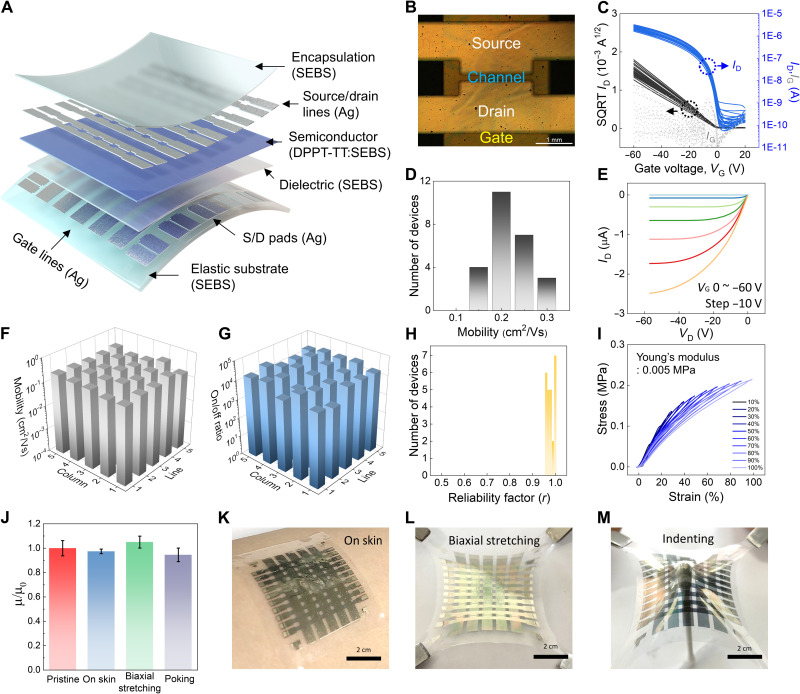
Skin-like organic transistor active-matrix array. (**A**) Schematic of the skin-like organic transistor active-matrix array structure. (**B**) OM image of a channel in the active-matrix array. (**C**) Transfer curves of all devices in the skin-like organic transistor active-matrix array. (**D**) Distribution of field-effect mobilities of the active-matrix array. (**E**) Typical output curve of a skin-like active-matrix organic transistor. (**F**) Three-dimensional mapping of mobility and (**G**) on/off current ratio of all pixel devices in the active-matrix array. (**H**) Distribution of reliability factor from all devices in stretchable active-matrix transistors. (**I**) Cyclic stress-strain curves of the skin-like organic transistor active-matrix array for extracting its Young’s modulus. (**J**) Mobilities of stretchable active-matrix transistors under various conditions. (**K**) Photographs of stretchable active matrix on human skin, (**L**) under biaxial stretching, and (**M**) under indenting.

For practical feasibility of the stretchable Ag metallization, we fabricated a fully stretchable strain-insensitive 5 × 5 active-matrix array of OTFTs, as shown in Fig. 4A. A specific OM image of a device in an active-matrix array is presented in Fig. 4B. All the stretchable OTFTs performed well, and the transfer characteristics and statistical distribution of the field-effect mobilities are presented in Fig. 4 (C and D, respectively). The typical output curve shown in Fig. 4E indicates that the Ag-metallized electrode forms an ohmic contact on the DPPT-TT/SEBS nanoconfined stretchable semiconductor in the active-matrix array. The values of the field-effect mobility and on/off ratio of the 25 OTFTs in the active-matrix array are mapped, as shown in Fig. 4 (F and G), and the results show that the OTFTs in the array exhibited low performance variability. All the transistors exhibited extremely high reliability factors (>0.96) as an indicator (1: ideal; 0: nonideal) representing the degree of overestimation and miscalculation of the mobility (Fig. 4H) (**42**, **43**)]. Moreover, the cyclic strain-stress characteristics of the active-matrix array were measured, as shown in Fig. 4I, and the results show that it is highly elastic up to 100% strain and has lower Young’s modulus (0.005 MPa) than that of human skin (10^−1^ MPa), which can significantly reduce the feeling of irritation caused by foreign matter on skin. These are essential mechanical properties for a device to be integrated on human skin. Last, the reliability of the active-matrix array under arbitrary deformations experienced on the human skin was estimated. Figure 4J presents the mobility changes of the active-matrix array under various deformations, such as attaching to curved skin (arm), biaxial stretching (30%), and even indenting (depth: 1 cm) with a cotton swab (Fig. 4, K to M, and fig. S50). In situ measurement of the active-matrix transistor during various deformations in series is shown in fig. S51 and movie S2. The electrical property of the active-matrix array preserved the initial field-effect mobility well under all the harsh conditions.

## DISCUSSION

In summary, we introduced Ag metallization of elastomeric semiconductors with excellent stretching endurance and adhesion durability for stretchable OTFTs. The metallization is based on the unique property of Ag atoms, which have high diffusivity as compared to other metals in a polymer matrix. Therefore, the nucleation and growth of a thermally evaporated Ag thin film occur from inside the elastomeric semiconductor, and the intermixing of the Ag nanoclusters and polymer chains at the interface occurs between the Ag thin film and elastomeric semiconductor film, which enhances the adhesion between them. In contrast to previously reported stretchable electrodes, our Ag-metallized electrodes on elastomeric semiconductor films exhibited high conductivity (>10^4^ S/cm). High conductivity was maintained, and no delamination was observed even when the Ag-metallized electrodes are subjected to 100% tensile strain for 10,000 stretching cycles and several adhesive tape tests. Owing to the enhanced adhesion, the Ag-metallized electrode released the applied external strain by forming nanoscale cracks, thus enabling it to exhibit excellent stretching endurance and adhesion durability under various deformations. In addition to the superior mechanical durability, it was found that Ag metallization forms Ag_2_O with a high work function (Φ = 5.2 eV) in the intermixing layer, which effectively lowers the hole injection barrier at the interface between the Ag-metallized electrode and the elastomeric semiconductor film. Last, to demonstrate the application potential of the stretchable Ag metallization in stretchable OTFTs, we fabricated a fully stretchable strain-insensitive 5 × 5 active-matrix OTFT array using the Ag metallization technique, and all the transistors in the array exhibited a highly stable electrical performance while being attached to curved human skin, biaxially stretched, and even severely indented. With our straightforward thermal evaporation–based strategy for preparing stretchable metallization, we believe that Ag metallization could aid in allowing stretchable OTFTs to progress toward the realization of skin-inspired electronics.

## MATERIALS AND METHODS

### Materials

Ag (silver, 99.99%, 3- to 5-mm granule), Au (gold, 99.99%, 3 mm–by–3 mm pellet), and Cu (copper, 99.99%, 3 mm–by–5 mm pellet) were purchased from SYSCIENCE. DPPT-TT was purchased from Derthon. Three types of SEBS were provided from AsahiKASEI. H1221 (S/EB weight ratio, 12/88), H1062 (S/EB weight ratio, 18/82), and H1052 (S/EB weight ratio, 20/80) were used as the elastic matrix of the semiconducting layer, elastic substrate, and dielectric layer, respectively. All chemicals were used as received without further purification.

### Stretchable metallization and characterization

The elastic substrates for stretchable metallization were prepared by casting SEBS H1062 (100 mg/ml in toluene) onto a slide glass. After drying for overnight, each metal was evaporated on elastic substrates in high vacuum (below 5.0 × 10^−6^ torr). The conductivity of the electrodes was measured using a four-probe meter (MITSUBISHI, MCP-T610). The work functions of each metal electrode were measured using a photoelectron spectroscope (RKI INSTRUMENTS, AC-2). The stretchable metallization was analyzed using an OM (Leica, DM4 M) for micromorphology, AFM (Bruker, Dimension 3100 SPM) for nanoscale morphology, XPS (Thermo Electron, K-Alpha) for atomic components, and high-resolution TEM and STEM with EDS mapping (JEOL, JEM ARM 200F NEOARM, at 200 kV) for nanostructure and atomic morphology. Cyclic stretching tests were performed using a handmade machine (stretching rate: 100 cm/min).

### Device fabrication and measurement

(i) For rigid substrate-based organic field-effect transistors (OFETs): The solution for the semiconductor was prepared by dissolving 1 weight % of DPPT-TT and SEBS H1221 with 1:9 weight ratio in anhydrous chlorobenzene at 120°C for overnight. For the fabrication of the semiconducting film, these solutions with filtration using a 0.2-μm Polytetrafluoroethylene-D filter were spin-coated on an octadecyltrimethoxysilane (OTS)–treated Si wafer with 300 nm of SiO_2_ layer at 1000 rpm for 1 min and then annealed at 180°C for 1 hour under a N_2_ atmosphere glove box [H_2_O < 0.01 parts per million (ppm) and O_2_ < 0.01 ppm]. The dielectric layer was prepared by spin-coating SEBS H1052 solution (60 mg/ml in toluene) onto an indium tin oxide (ITO; gate electrode; sheet resistance, 20 ohm/square) glass substrate. The semiconducting films were then transferred using a PDMS stamp from the OTS-Si wafer onto the SEBS/ITO glass substrate. Last, source and drain electrode with different metals were evaporated using a thermal evaporator (*W*: 1000 μm; *L*: 150 μm). Figure S1 shows the schematic illustration of the device fabrication in detail. For stabilization of the transferred films, all devices were aged overnight in an autodry desiccator after metal evaporation. All electrical characteristics of OFET were measured using a probe station connected to a KEITHLEY 4200 under ambient condition. (ii) For fully stretchable OFET and active-matrix transistor array: A stretchable Ag (50 nm) gate electrode was prepared on the elastic substrate by thermal evaporating under high vacuum (below 5.0 × 10^−6^ torr). The spin-coated dielectric layer (1.5 μm) and semiconducting thin-film (90 nm) on the OTS-treated Si wafer were transfer-printed on the Ag (gate electrode)–deposited elastic substrate. Then, source and drain electrodes were thermally evaporated on the elastic semiconducting film. The changed capacitances of the stretchable SEBS dielectric according to applied strain are summarized in tables S2 and S3. The strain-stress curves of the fully stretchable active-matrix transistor array were obtained by a force tester (AND, MCT-2150; strain rate: 100 cm/min).
