# Supplementary material for: Mechanically robust stretchable semiconductor metallization for skin-inspired organic transistors
Source: Sci Adv. 2022 Dec 21;8(51):eade2988. doi: 10.1126/sciadv.ade2988 (PMC9770969; doi:10.1126/sciadv.ade2988)
Supplement: Supplementary file 1 — Figs. S1 to S51 Tables S1 to S3 [file sciadv.ade2988_sm.pdf]

Science Advances
AAAS

Supplementary Materials for

Mechanically robust stretchable semiconductor metallization for skin-inspired organic transistors

Min Hyouk Kim *et al.*

Corresponding author: Tae Il Lee, t2.lee77@gachon.ac.kr; Jin Young Oh, jyoh@khu.ac.kr

Sci. Adv. **8**, eade2988 (2022)
DOI: 10.1126/sciadv.ade2988

The PDF file includes:

Figs. S1 to S51
Tables S1 to S3
Legends for movies S1 and S2

Other Supplementary Material for this manuscript includes the following:

Movies S1 and S2

This PDF file includes:

Fig. S1. Fabrication procedure of organic field-effect transistors (OFETs).

Fig. S2. Contact resistance measurement of the OFETs with Ag electrode

Fig. S3. Contact resistance measurement of the OFETs with Au electrode.

Fig. S4. Contact resistance comparison of OFETs with Ag, Au and Cu electrodes.

Fig. S5. Schottky barrier measurement of OFETs with Ag and Au electrodes.

Fig. S6. Photoelectron spectra of Ag, Au and DPP-SEBS film in air.

Fig. S7. XPS spectra Ag 3d peak as a function of etching time.

Fig. S8. XPS spectra with silver oxide peak as a function of etching time.

Fig. S9. Electrical conductivity of Ag film on DPPT-TT:SEBS and SEBS films according to Ag metallization thickness.

Fig. S10. Resistance changes of the Ag metallization on stretchable semiconductor according to aging time in air and glove box.

Fig. S11. Optical microscope images of Ag metallization on DPPT-TT:SEBS as a function of its thickness on various strains.

Fig. S12. Optical microscope images of Au metallization on DPPT-TT:SEBS as a function of its thickness on various strains.

Fig. S13. Optical microscope images of Cu metallization on DPPT-TT:SEBS as a function of its thickness on various strains.

Fig. S14. Optical microscope images of Ag metallized DPPT-TT:SEBS film as a function of its thickness with marked crack area under strain.

Fig. S15. Optical microscope images of Au metallized DPPT-TT:SEBS film as a function of its thickness with marked crack area under strain.

Fig. S16. Optical microscope images of Cu metallized DPPT-TT:SEBS film as a function of its thickness with marked crack area under strain.

Fig. S17. Crack area percent of each thickness Ag, Au and Cu metallized films on various strains calculated from optical microscope images.

Fig. S18. The resistance of each metal electrode with various thickness on different strain.

Fig. S19. Optical microscope images of Ag metallization on DPPT-TT:SEBS as a function of evaporation rate on various strains.

Fig. S20. Optical microscope images of Ag metallized DPPT-TT:SEBS film as a function of evaporation rate with marked crack area under strain.

Fig. S21. Optical microscope images of Au metallization on DPPT-TT:SEBS as a function of evaporation rate on various strains.

Fig. S22. Optical microscope images of Au metallized DPPT-TT:SEBS film as a function of evaporation rate with marked crack area under strain.

Fig. S23. Optical microscope images of Cu metallization on DPPT-TT:SEBS as a function of evaporation rate on various strains.

Fig. S24. Optical microscope images of Cu metallized DPPT-TT:SEBS film as a function of evaporation rate with marked crack area under strain.

Fig. S25. Crack area percent of each evaporation rate Ag, Au and Cu metallized films on various strains calculated from optical microscope images.

Fig. S26. XPS depth profiling analysis with Au and Cu electrode on DPPT-TT:SEBS film.

Fig. S27. Deformation of stretchable semiconductor metallization during durability test.

Fig. S28. Adhesion force measurement of stretchable semiconductor metallization.

Fig. S29. Optical microscope images of 50nm of Ag metallized DPPT-TT:SEBS film during cyclic adhesive-tape tests.

Fig. S30. Morphology analysis of stretchable semiconductor metallization using AFM.

Fig. S31. STEM analysis of Ag metallized DPPT-TT:SEBS film.

Fig. S32. STEM analysis of Ag metallized DPPT-TT:SEBS film under 50% strain.

Fig. S33. STEM analysis of Ag metallized DPPT-TT:SEBS film under 100% strain.

Fig. S34. Cross-section TEM analysis of Ag metallized DPPT-TT:SEBS film.

Fig. S35. Fabrication procedure of fully stretchable organic transistor.

Fig. S36. Transfer curve of OTFT device with Ag electrodes and estimating electrical working property during drain voltage sweep.

Fig. S37. Transfer and output curves of fully stretchable transistors fabricated with Ag metallization during 100 % stretching cycle according to stretching direction.

Fig. S38. Transfer and output curves of fully stretchable transistors fabricated with Ag metallization during multiple stretching cycles to channel length direction at 25% strain up to 10,000 times.

Fig. S39. Transfer and output curves of fully stretchable transistors fabricated with Ag metallization during multiple stretching cycles to channel width direction at 25% strain up to 10,000 times.

Fig. S40. Transfer and output curves of fully stretchable transistors fabricated with Ag metallization during multiple stretching cycles to channel length direction at 50% strain up to 10,000 times.

Fig. S41. Transfer and output curves of fully stretchable transistors fabricated with Ag metallization during multiple stretching cycles to channel width direction at 50% strain up to 10,000 times.

Fig. S42. Transfer and output curves of fully stretchable transistors fabricated with Ag metallization during multiple stretching cycles to channel length direction at 75% strain up to 10,000 times.

Fig. S43. Transfer and output curves of fully stretchable transistors fabricated with Ag metallization during multiple stretching cycles to channel width direction at 75% strain up to 10,000 times.

Fig. S44. Transfer and output curves of fully stretchable transistors fabricated with Ag metallization during multiple stretching cycles to channel length direction at 100% strain up to 10,000 times.

Fig. S45. Transfer and output curves of fully stretchable transistors fabricated with Ag metallization during multiple stretching cycles to channel width direction at 100% strain up to 10,000 times.

Fig. S46. Biaxial stretching test of stretchable metallized OTFTs.

Fig. S47. Expandability test of stretchable metallization for other polymer semiconductor in terms of stretchability.

Fig. S48. Expandability test of stretchable metallization for other polymer semiconductor in terms of device performance.

Fig. S49. Expandability test of stretchable metallization for other polymer semiconductor in terms of adhesion.

Fig. S50. Transfer curve characteristics of fully stretchable organic transistor active-matrix array fabricated with Ag metallization under various deformations.

Fig. S51. In-situ measured transfer curve characteristics of fully stretchable organic transistor active-matrix array in Movie S2 under various deformations.

Table S1. Performance comparison of stretchable OTFTs and the specific information.

Table S2. Capacitance change of SEBS dielectric according to applied strain and stretching direction.

Table S3. Device geometry and capacitance changes of SEBS dielectric on biaxial stretching.

Other Supplementary Material for this manuscript includes the following:

Movie S1. In-situ measurement of conductivity of the stretchable metallized film before and after peel-off test using 3M tape (adhesion force: 2.2 N/cm).

Movie S2. In-situ measurement of the stretchable metallized OTFT active-matrix array for on-skin, biaxial stretching and indenting motions.

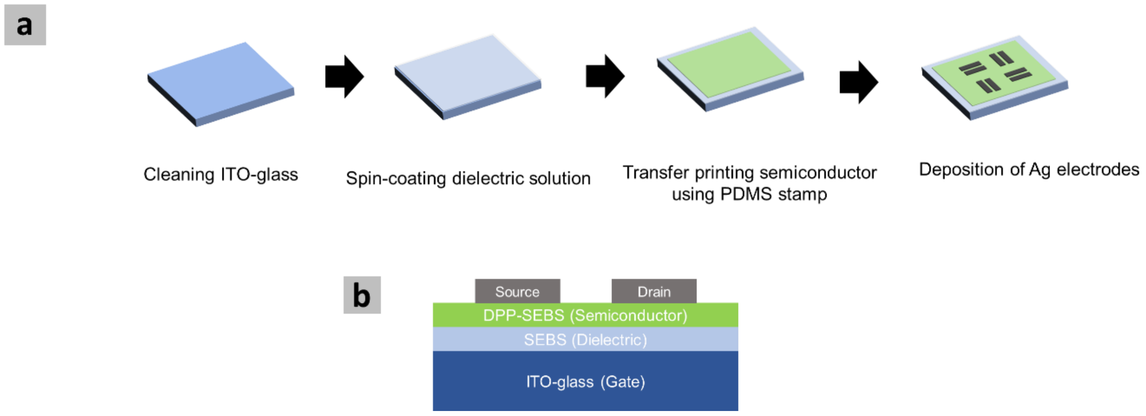

Fig. S1. Fabrication procedure of organic field-effect transistors (OFETs). (a) Fabrication procedure of OFET on ITO-glass for electrical characterization with different metal electrode. (b) Schematic image of structure ITO-glass OFET

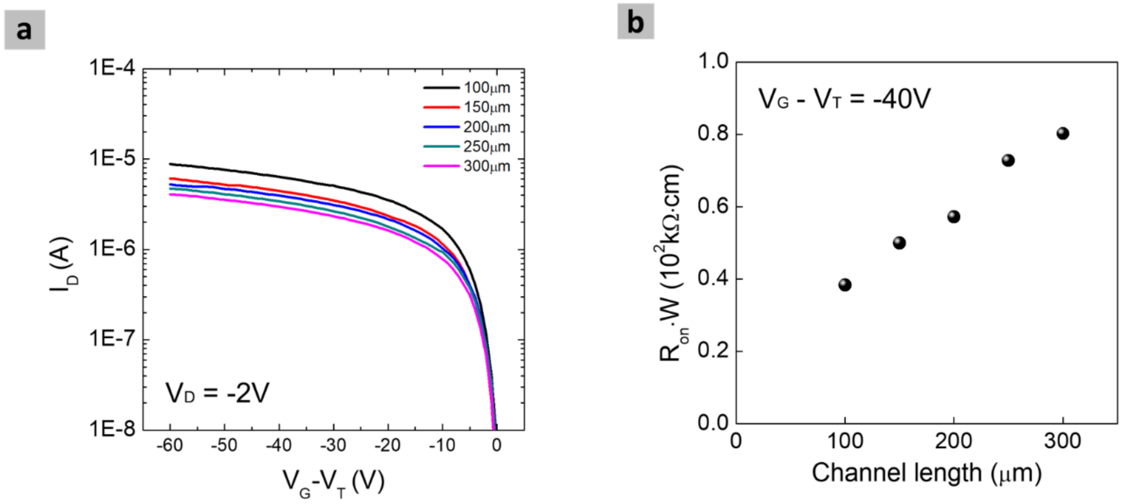

Fig. S2. Contact resistance measurement of the OFETs with Ag electrode (a) Drain current of OFET device with different channel length as a function of V_G-V_T. (b) Resistance change of OFET with Ag electrode as a function of channel length to calculate contact resistance

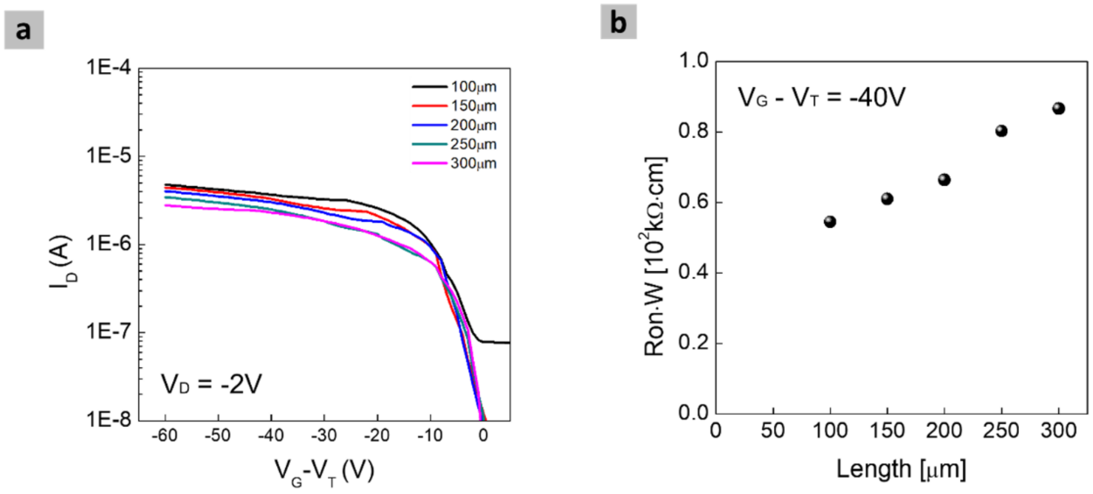

Fig. S3. Contact resistance measurement of the OFETs with Au electrode. (a) Drain current of OFETs with different channel length as a function of V_G-V_T. (b) Resistance change of OFETs with Au electrode as a function of channel length to calculate contact resistance

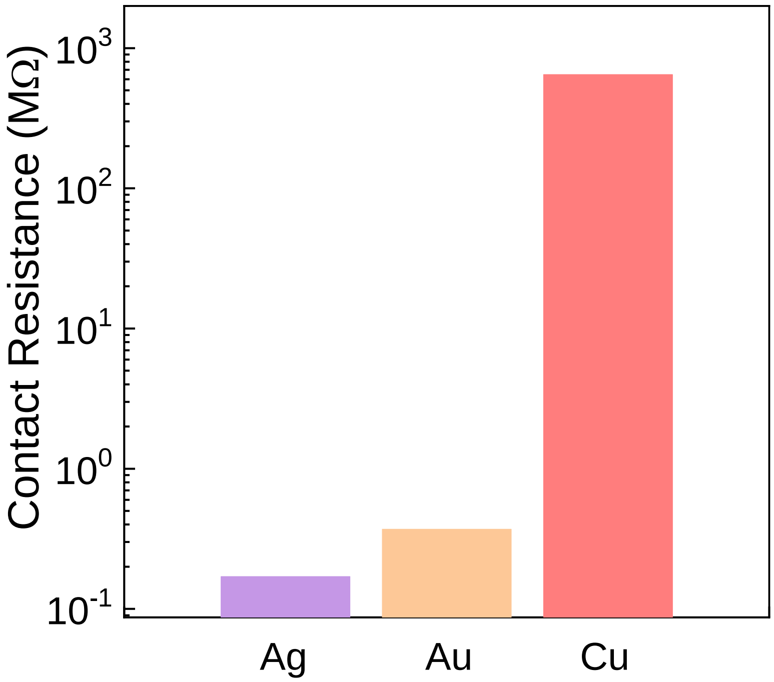

Fig. S4. Contact resistance comparison of OFETs with Ag, Au and Cu electrodes.

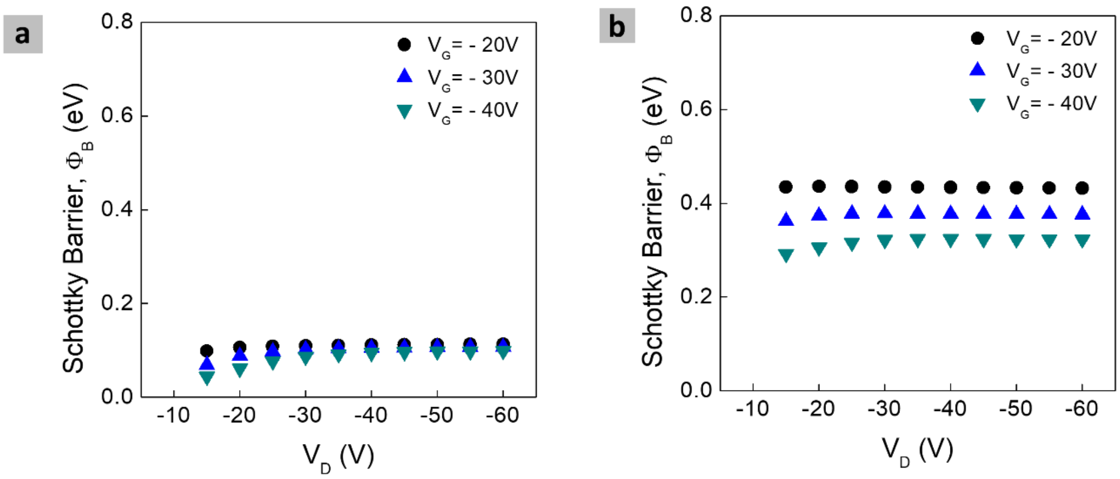

Fig. S5. Schottky barrier measurement of OFETs with Ag and Au electrodes. Calculated Schottky barrier of OFET with (a) Ag and (b) Au electrode as function of drain voltage and gate voltage.

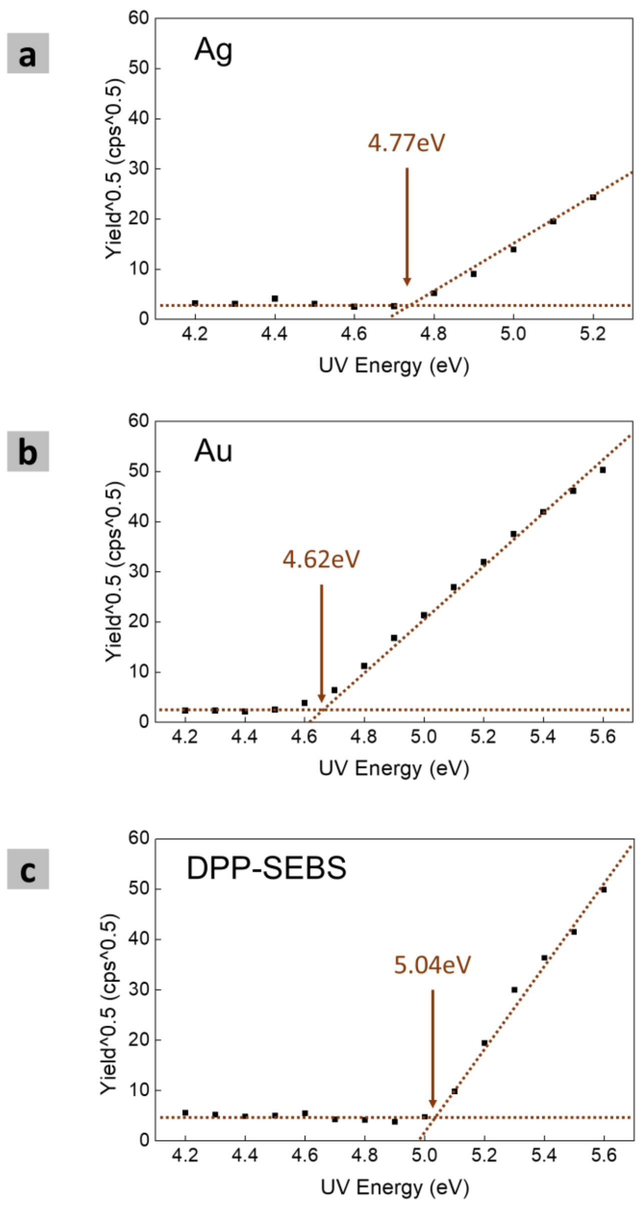

Fig. S6. Photoelectron spectra of (a) Ag (b) Au and (c) DPP-SEBS film in air. Arrow indicating the work function of each metal.

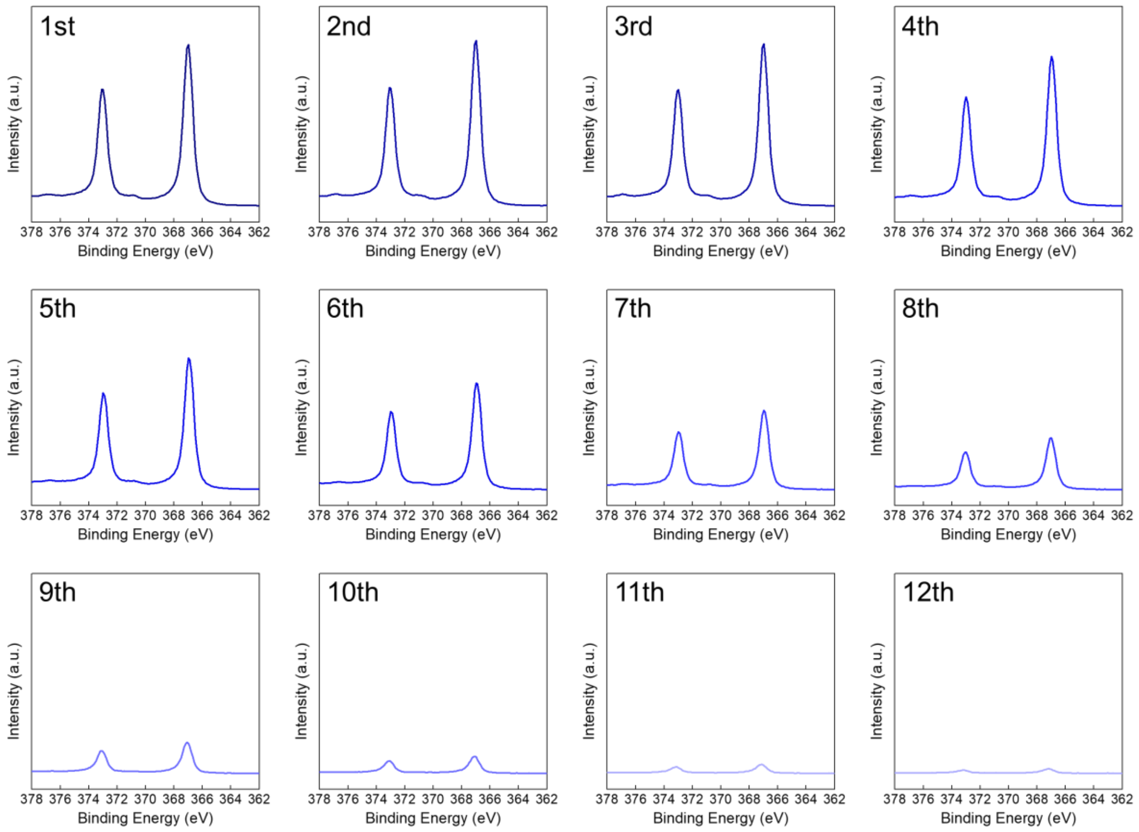

Fig. S7. XPS spectra in range from 360 to 376eV, showing Ag 3d peak, with different etching time.

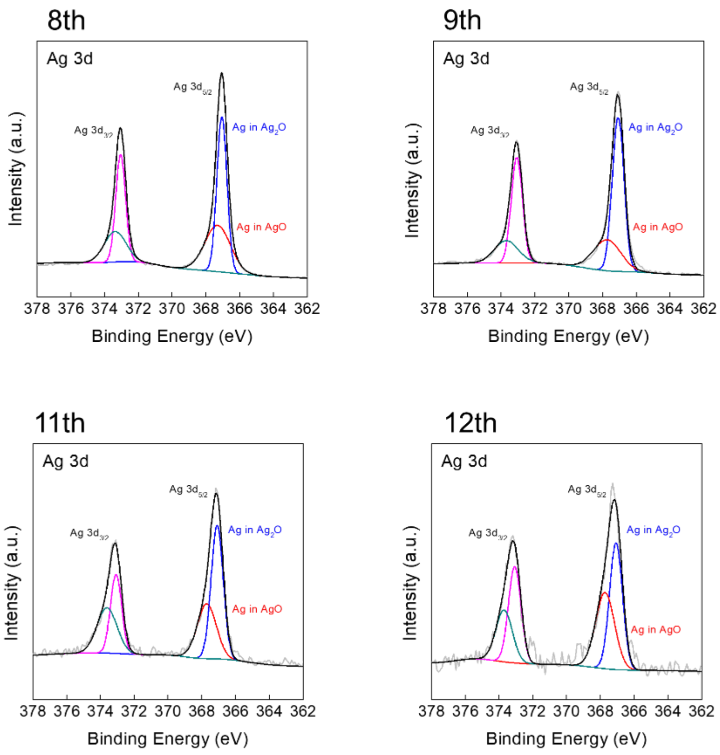

Fig. S8. XPS spectra with silver oxide peak in range from 360 to 376eV, showing Ag 3d peak, with different etching time.

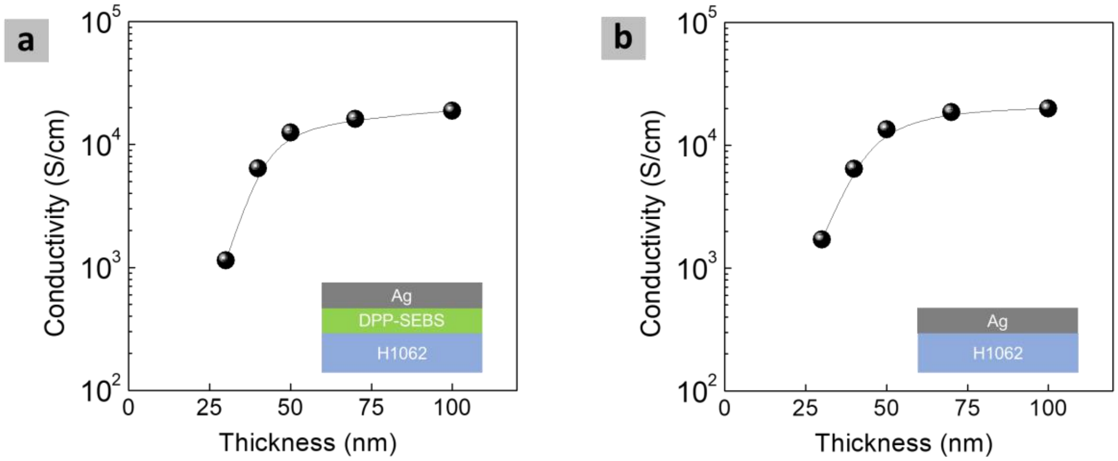

Fig. S9. Electrical conductivity of Ag film on (a) DPP-SEBS and (b) SEBS substrate as a function of Ag film thickness

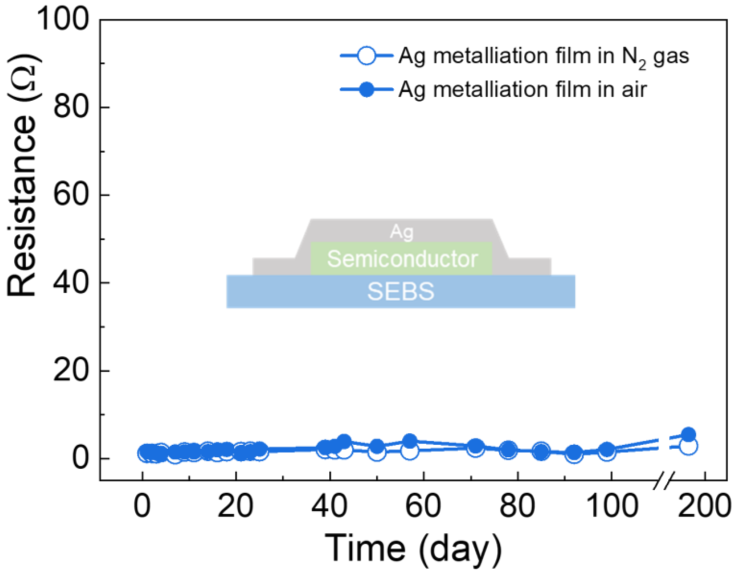

Fig. S10. Resistance changes of the Ag metallization on stretchable semiconductor according to aging time in air and glove box.

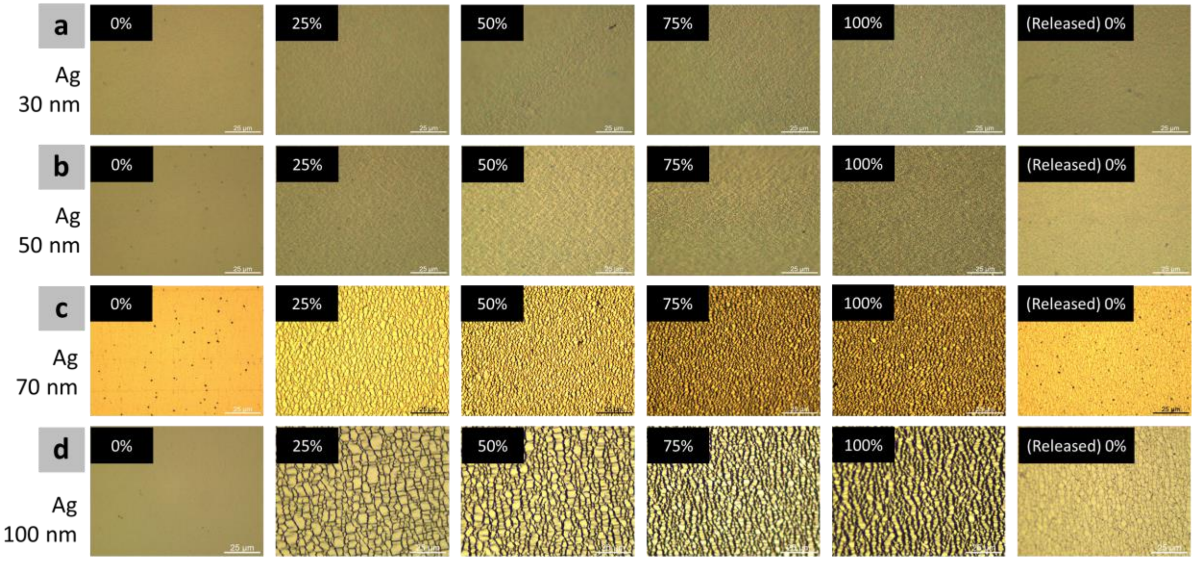

Fig. S11. Optical microscope images of (a) 30nm, (b) 50nm, (c) 70nm and (d) 100nm of Ag metallized films on DPPT-TT:SEBS semiconducting layer with different thickness and strain. (scale bar : 25μm)

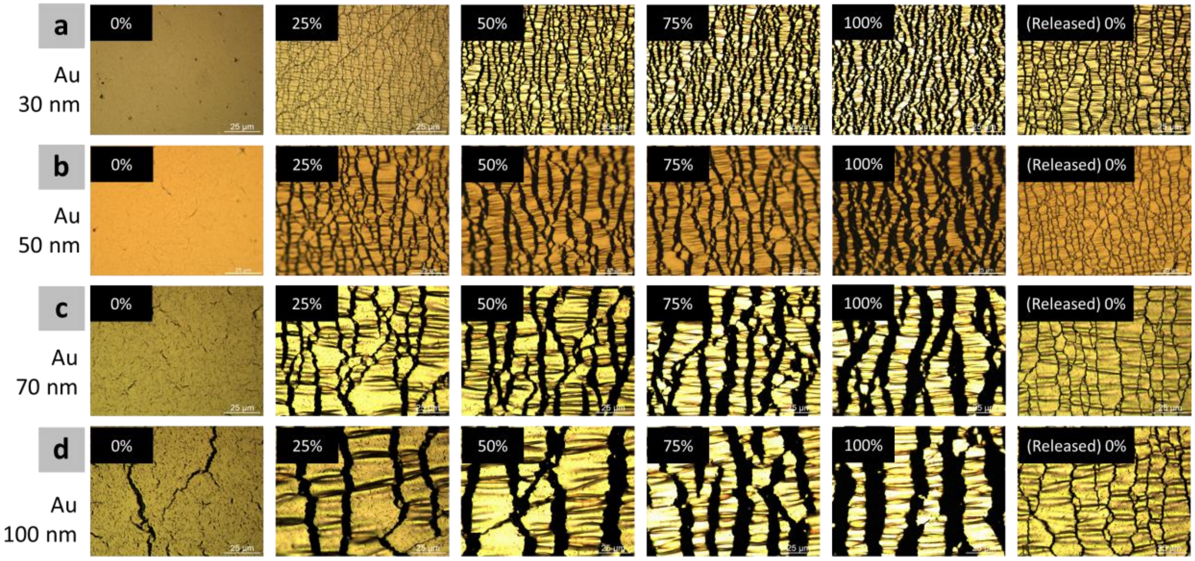

Fig. S12. Optical microscope images of (a) 30nm, (b) 50nm, (c) 70nm and (d) 100nm of Au film on DPPT-TT:SEBS semiconducting layer on different strain. (scale bar : 25μm)

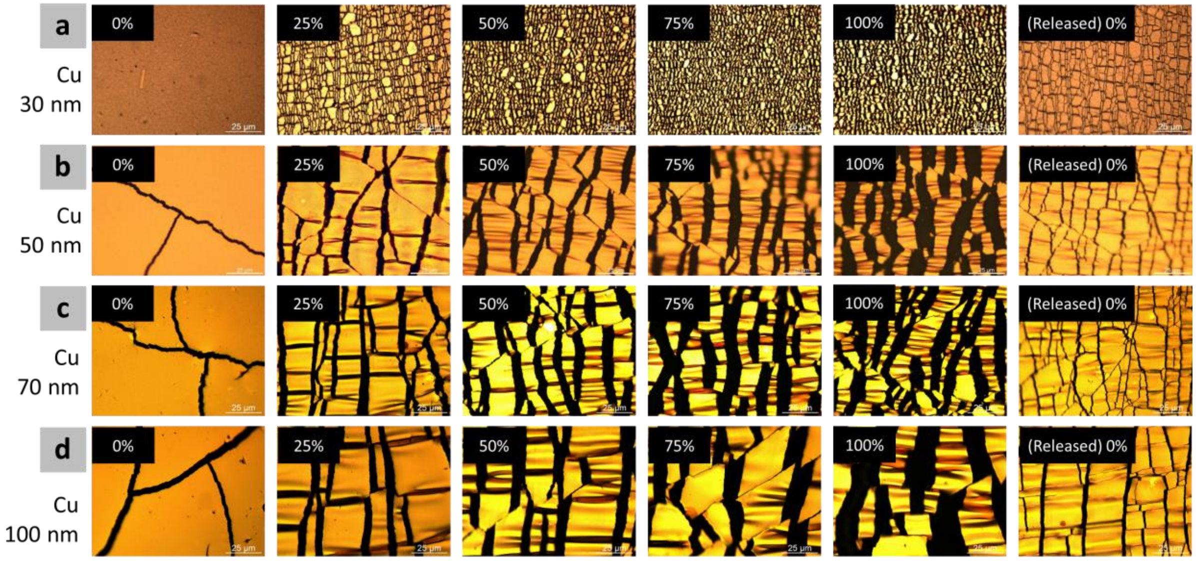

Fig. S13. Optical microscope images of (a) 30nm, (b) 50nm, (c) 70nm and (d) 100nm of Cu film on DPPT-TT:SEBS semiconducting layer on different strains. (scale bar : 25μm)

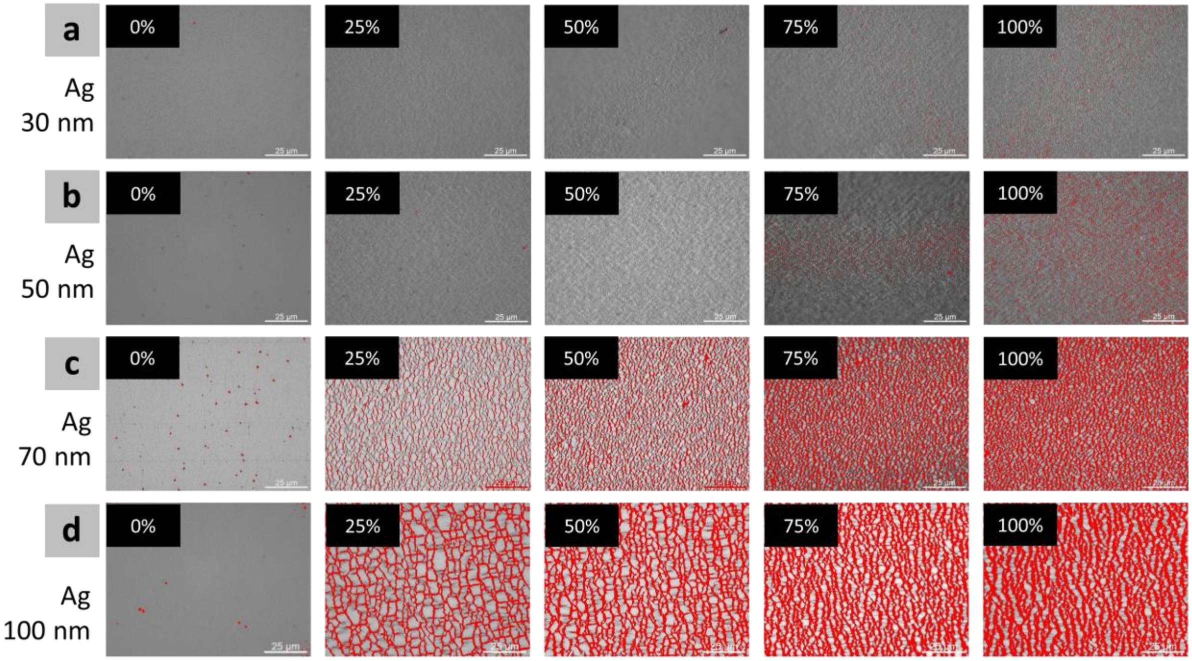

Fig. S14. Crack-area formed by various strain (0% ~ 100%) with (a) 30nm, (b) 50nm, (c) 70nm, (d) 100nm Ag electrode thickness.

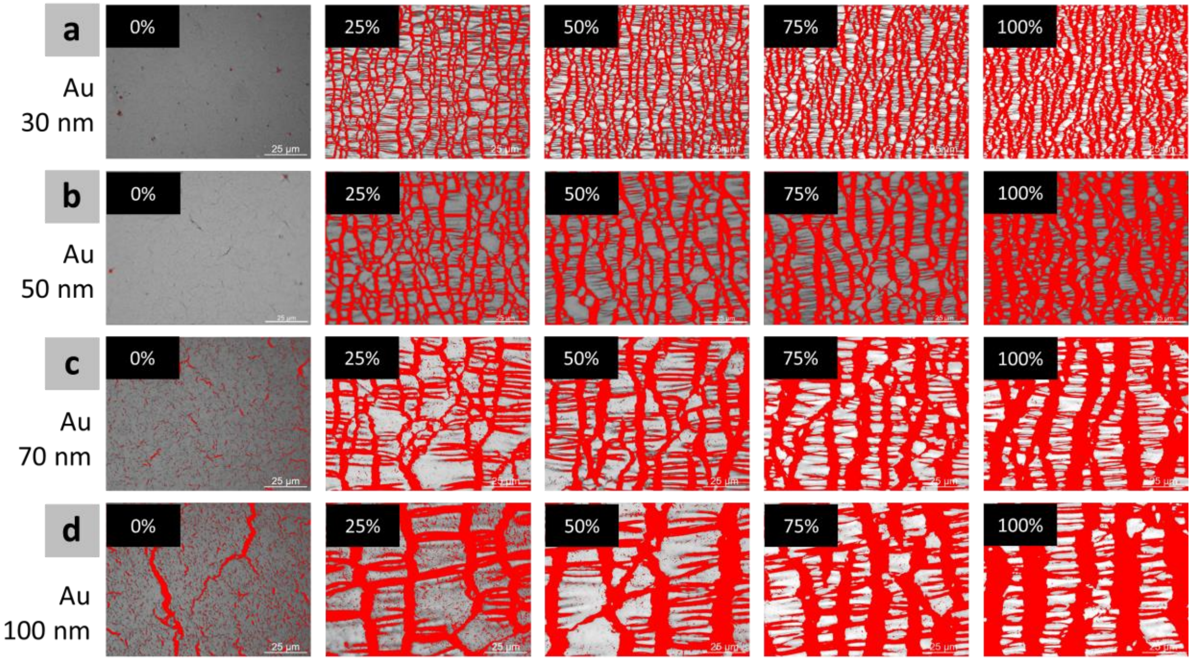

Fig. S15. Crack-area formed by various strain (0% ~ 100%) with (a) 30nm, (b) 50nm, (c) 70nm, (d) 100nm Au electrode thickness.

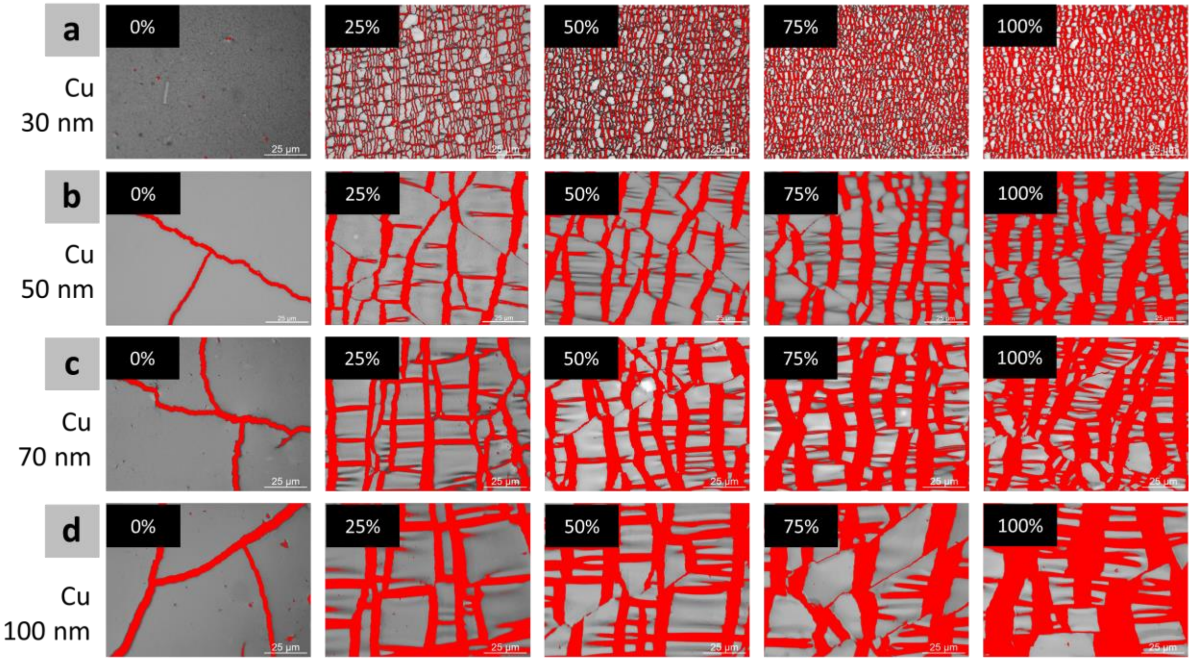

Fig. S16. Crack-area formed by various strain (0% ~ 100%) with (a) 30nm, (b) 50nm, (c) 70nm, (d) 100nm Cu electrode thickness.

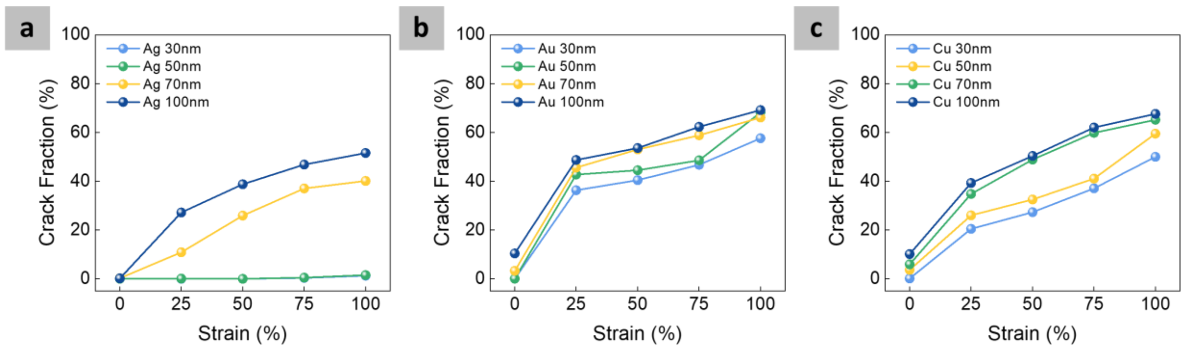

Fig. S17. Calculated crack area fraction from optical microscope images (Figure S13–S15) of (a) Ag, (b) Au and (c) Cu electrode with 30 - 100 nm thickness on different strain.

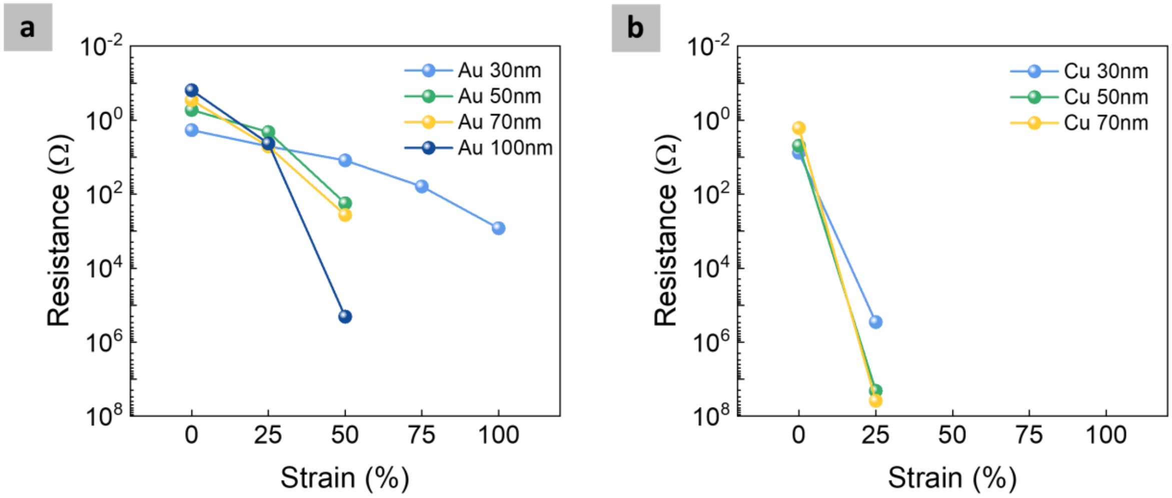

Fig. S18. The resistance of each metallized films with 30 - 100 nm thickness on different strain.

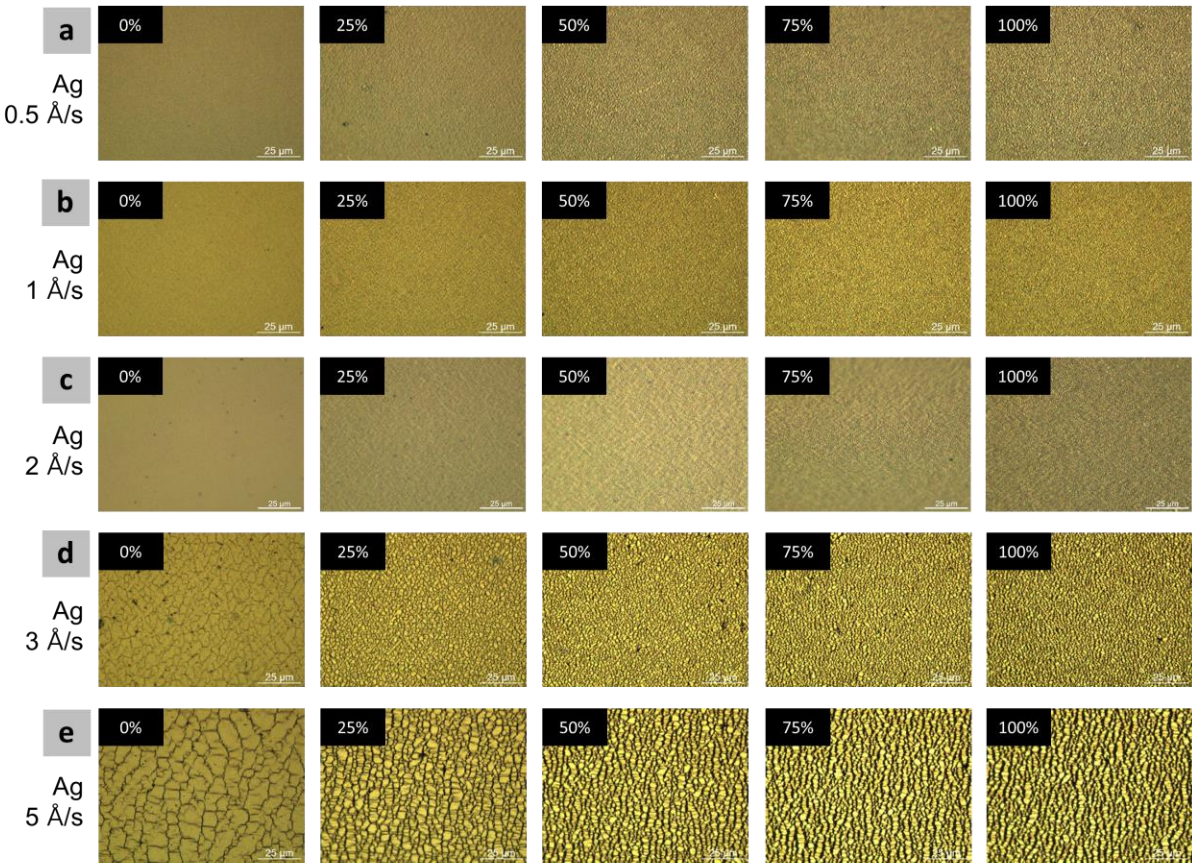

Fig. S19. Optical microscope images of 50nm of Ag film on DPPT-TT:SEBS deposited with (a) 0.5 Å/s, (b) 1 Å/s, (c) 2 Å/s, (d) 3 Å/s and (e) 5 Å/s evaporation rate. (scale bar : 25μm)

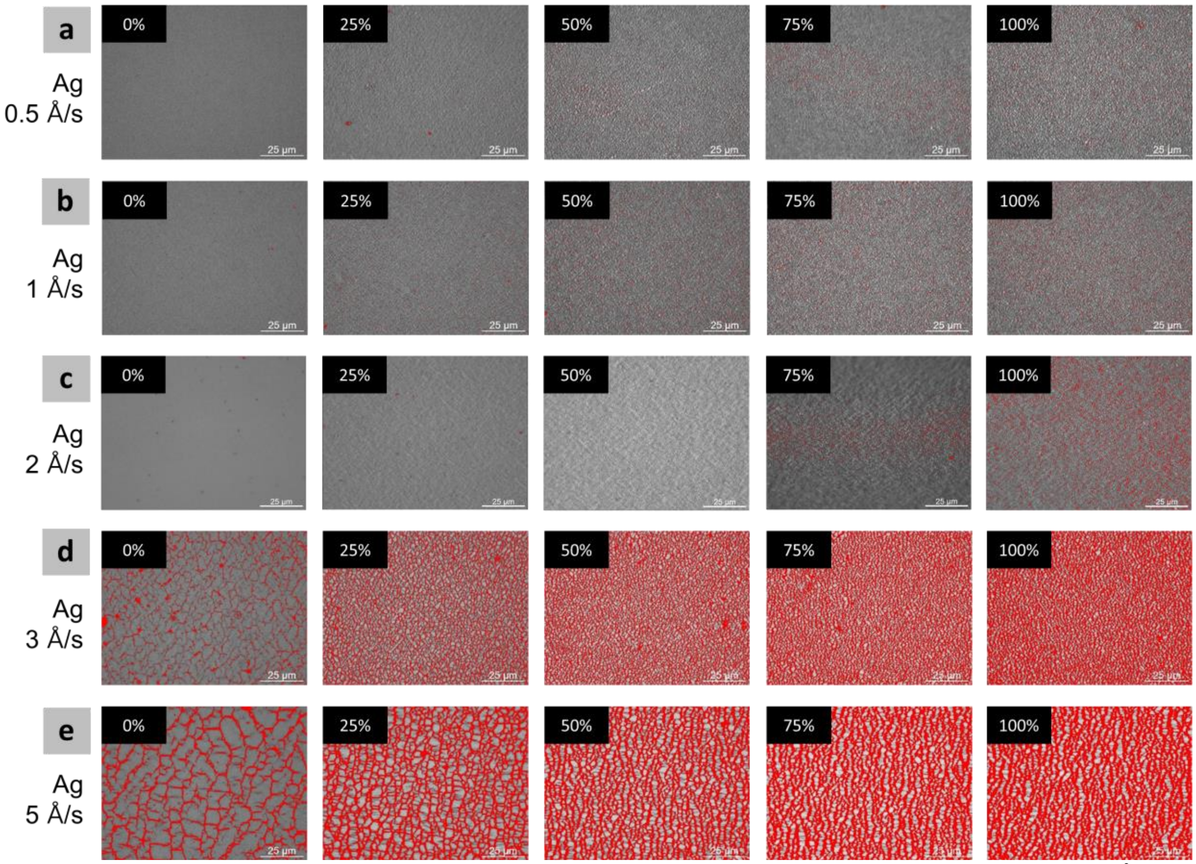

Fig. S20. Crack-area is expressed by red mark and each Ag film is deposited by (a) 0.5 Å/s, (b) 1 Å/s, (c) 2 Å/s, (d) 3 Å/s and (e) 5 Å/s evaporation rate. (scale bar : 25μm)

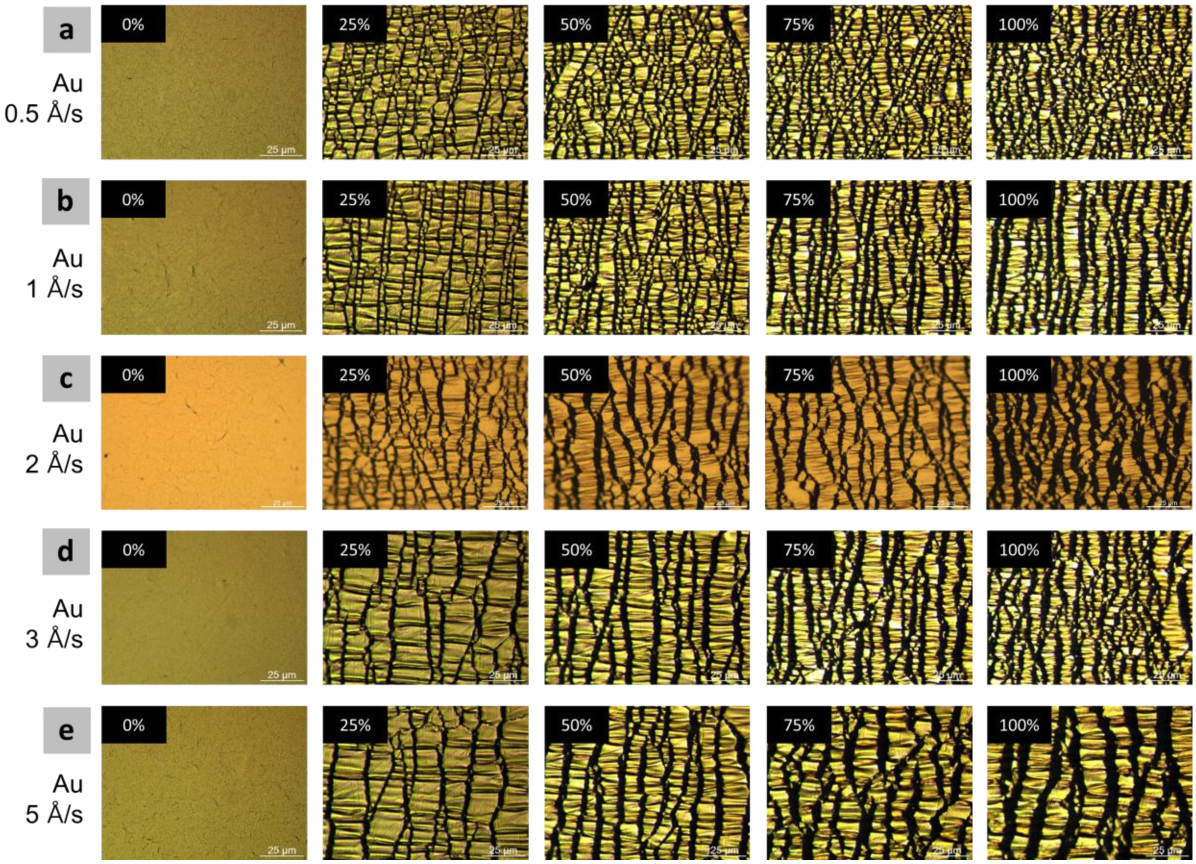

Fig. S21. Optical microscope images of 50nm of Au film on DPPT-TT:SEBS deposited with (a) 0.5 Å/s, (b) 1 Å/s, (c) 2 Å/s, (d) 3 Å/s and (e) 5 Å/s evaporation rate. (scale bar : 25µm)

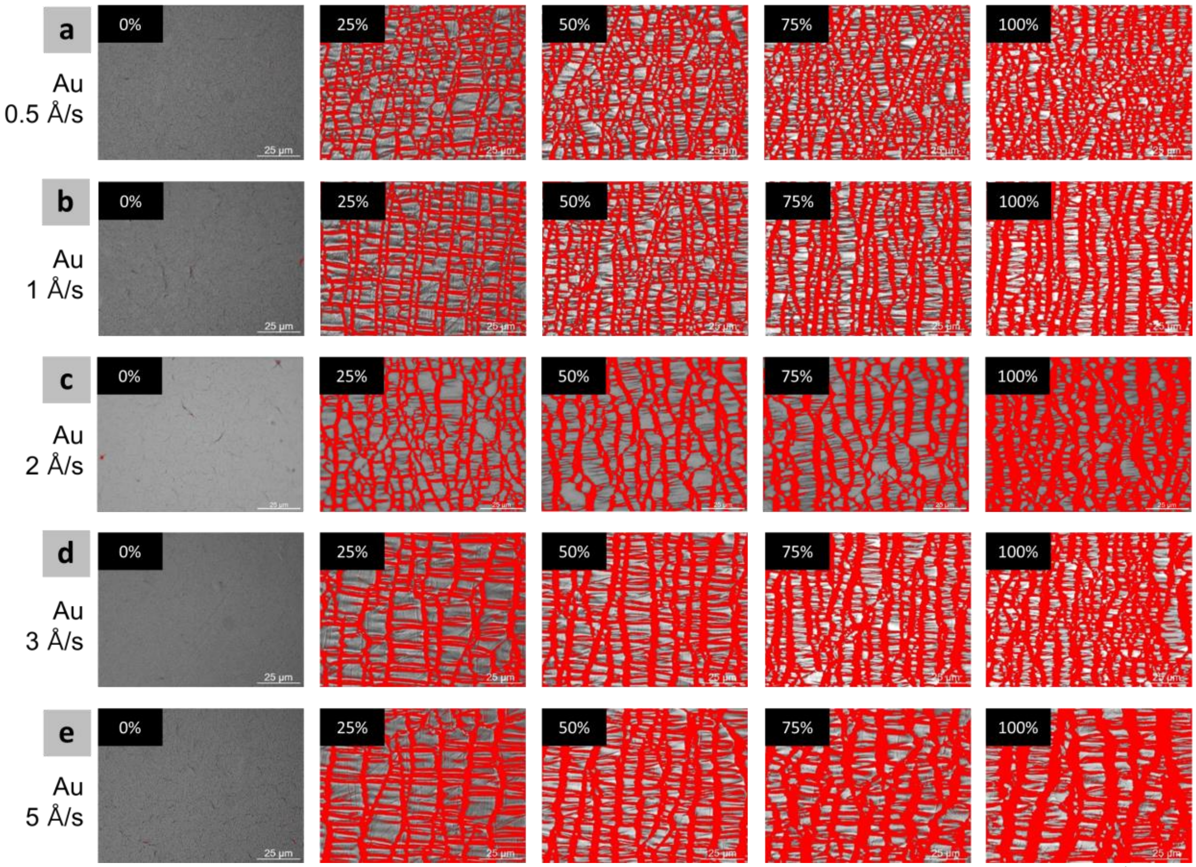

Fig. S22. Crack-area is expressed by red mark and each Au film is deposited by (a) 0.5 Å/s, (b) 1 Å/s, (c) 2 Å/s, (d) 3 Å/s and (e) 5 Å/s evaporation rate. (scale bar : 25μm)

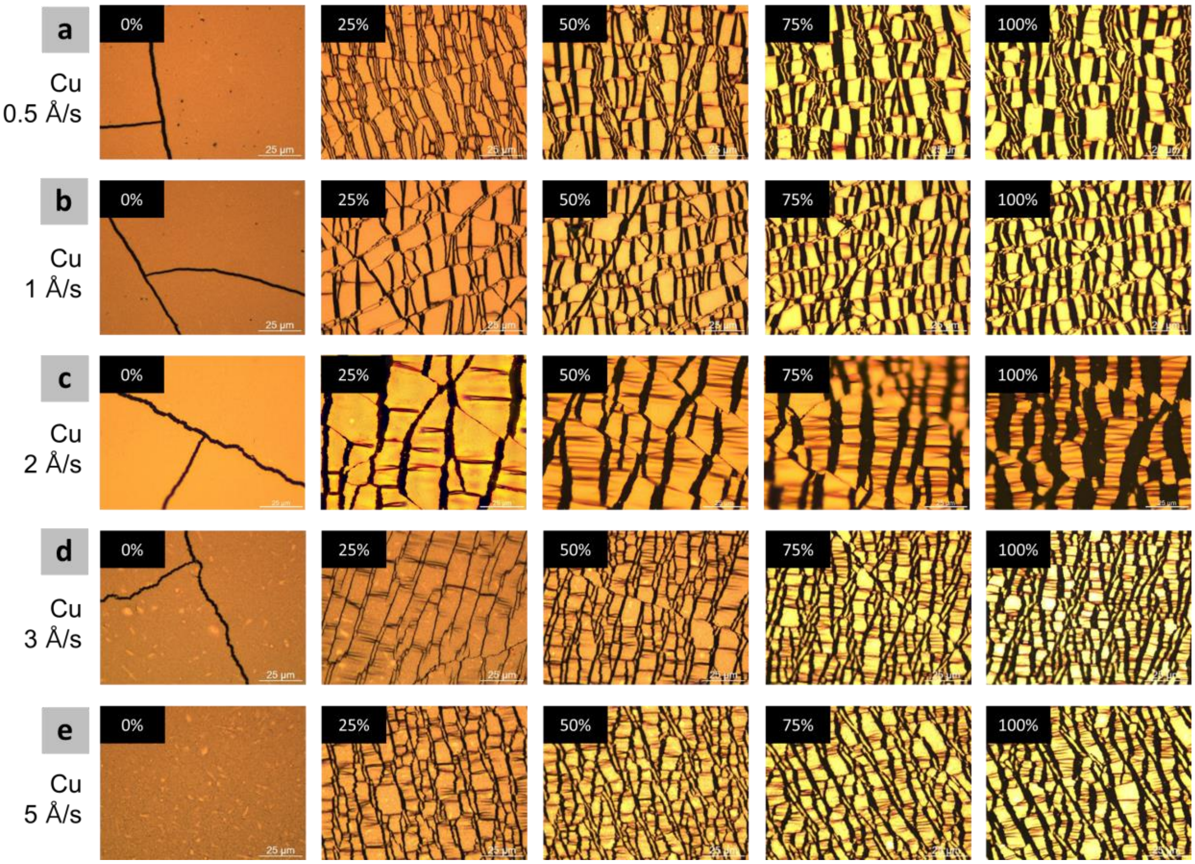

Fig. S23. Optical microscope images of 50nm of Cu film on DPPT-TT:SEBS deposited with (a) 0.5 Å/s, (b) 1 Å/s, (c) 2 Å/s, (d) 3 Å/s and (e) 5 Å/s evaporation rate. (scale bar : 25µm)

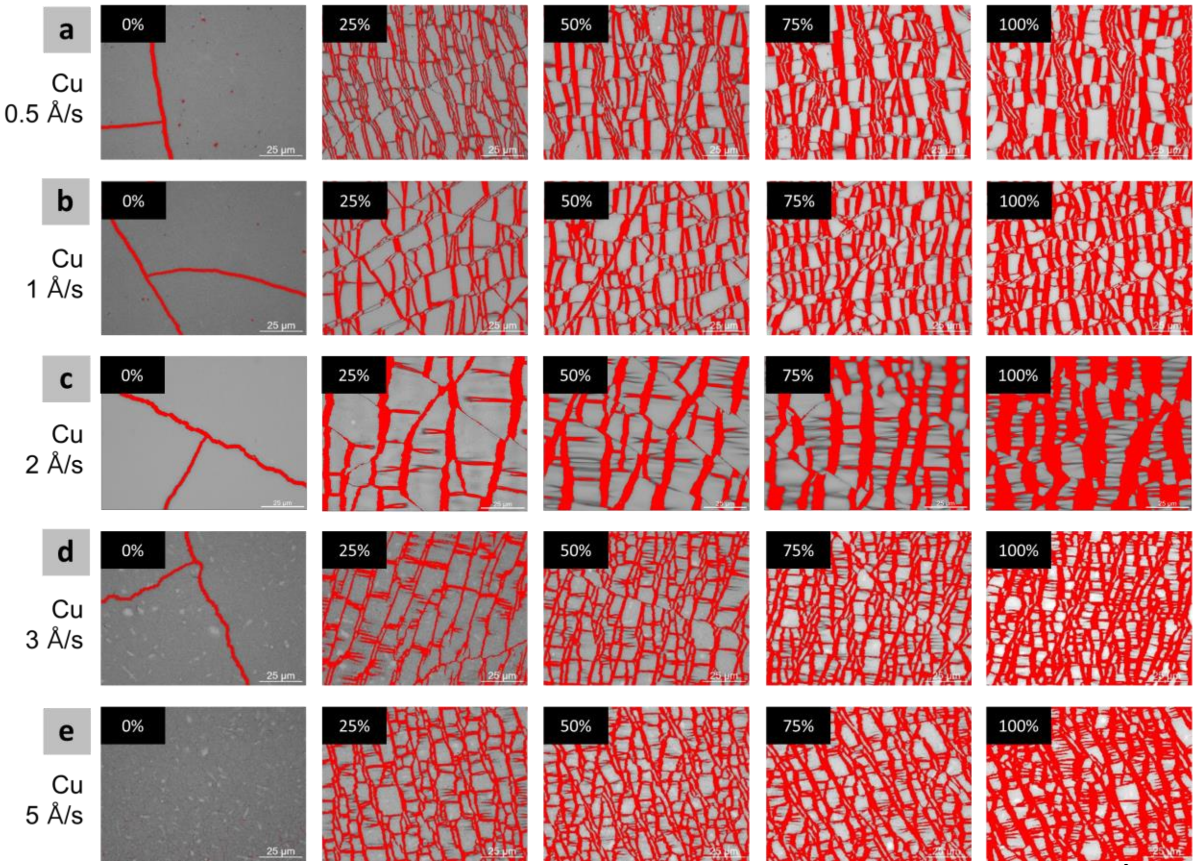

Fig. S24. Crack-area is expressed by red mark and each Cu film is deposited by (a) 0.5 Å/s, (b) 1 Å/s, (c) 2 Å/s, (d) 3 Å/s and (e) 5 Å/s evaporation rate. (scale bar : 25μm)

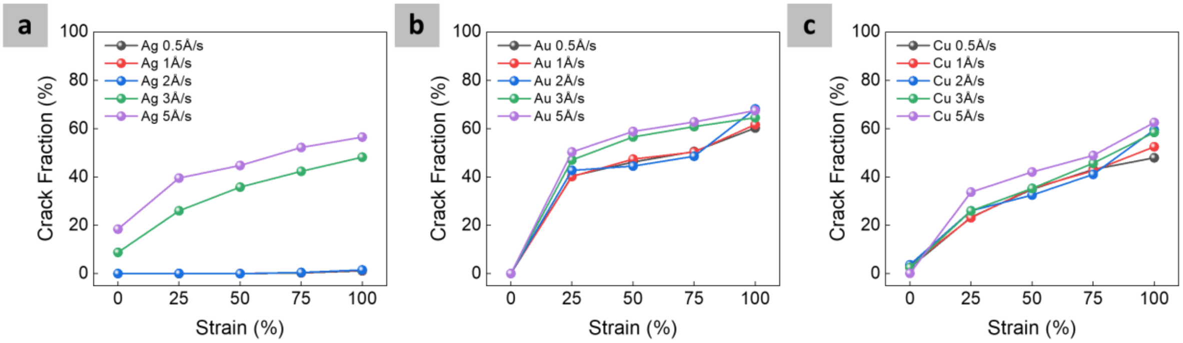

Fig. S25. Calculated crack area fraction from optical microscope images (Figure S21-S23) of (a) Ag, (b) Au and (c) Cu electrode with different strain and evaporation rate.

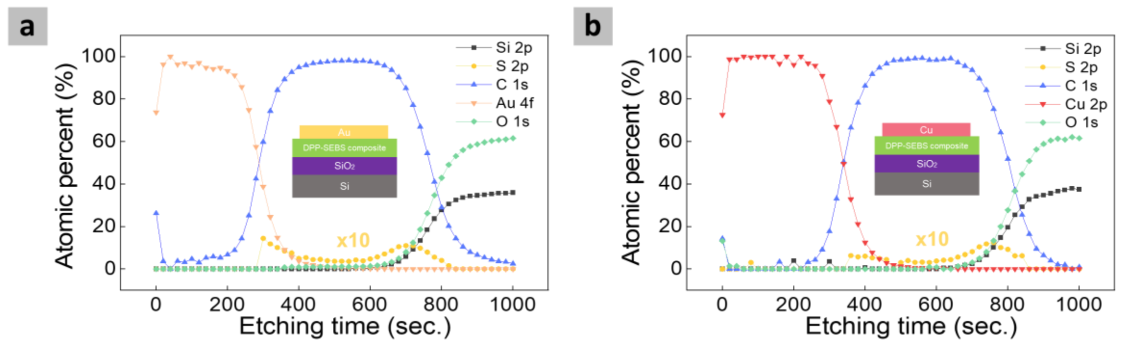

Fig. S26. XPS depth profiling analysis with (a)Au and (b) Cu electrode on stretchable semiconducting film to confirm metal permeation.

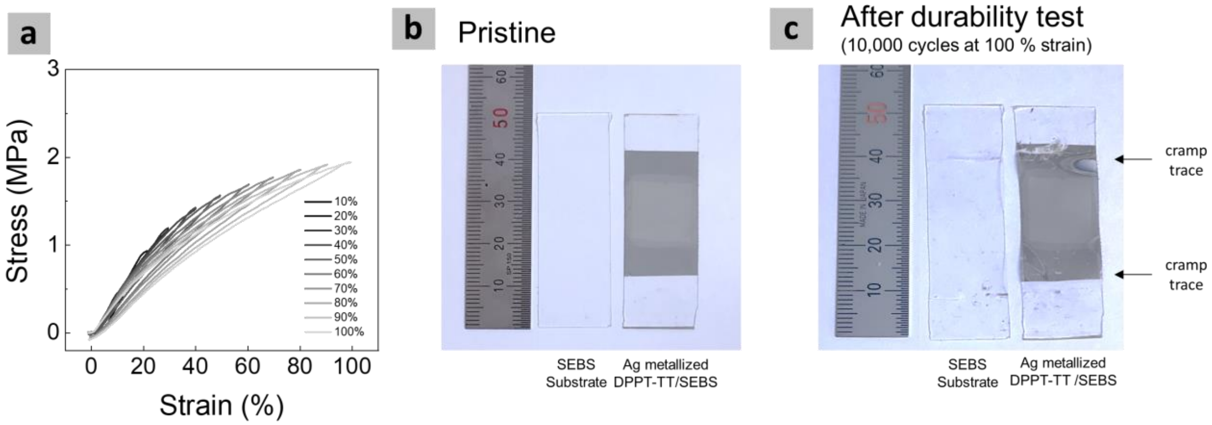

Fig. S27. Deformation of stretchable semiconductor metallization during durability test. (a) Strain-stress curves of the Ag metallized film on stretchable semiconductor/SEBS substrate. Photographs of SEBS substrate and Ag metallized semiconducting film on SEBS substrate (b) before and (c) after stretching test (10,000 cycles at 100 % strain, stretching rate: 35 mm/sec).

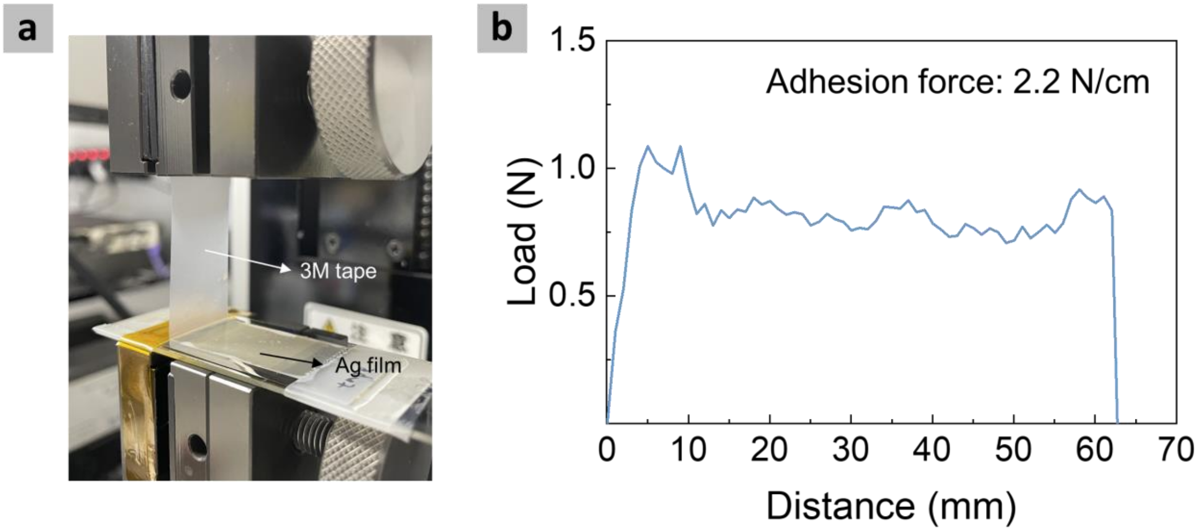

Fig. S28. Adhesion force measurement. (a) Force meter and calculate the adhesion force with (b) Load-Distance curve.

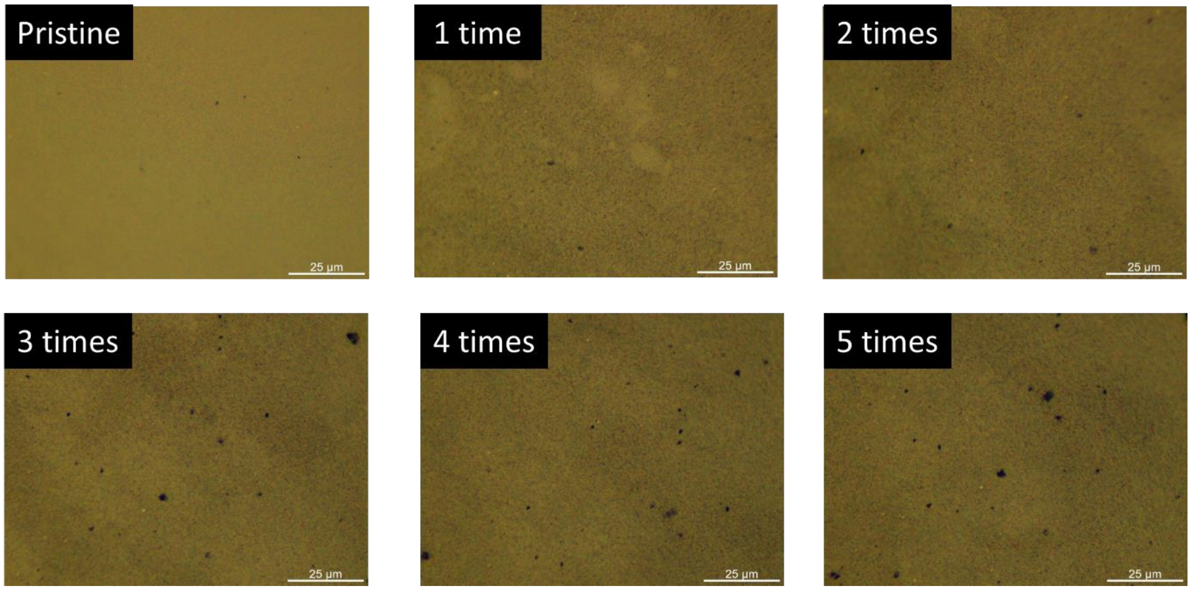

Fig. S29. Optical microscope images of 50nm of Ag metallized DPPT-TT:SEBS semiconducting film during cyclic adhesive-tape tests. (scale bar : 25μm)

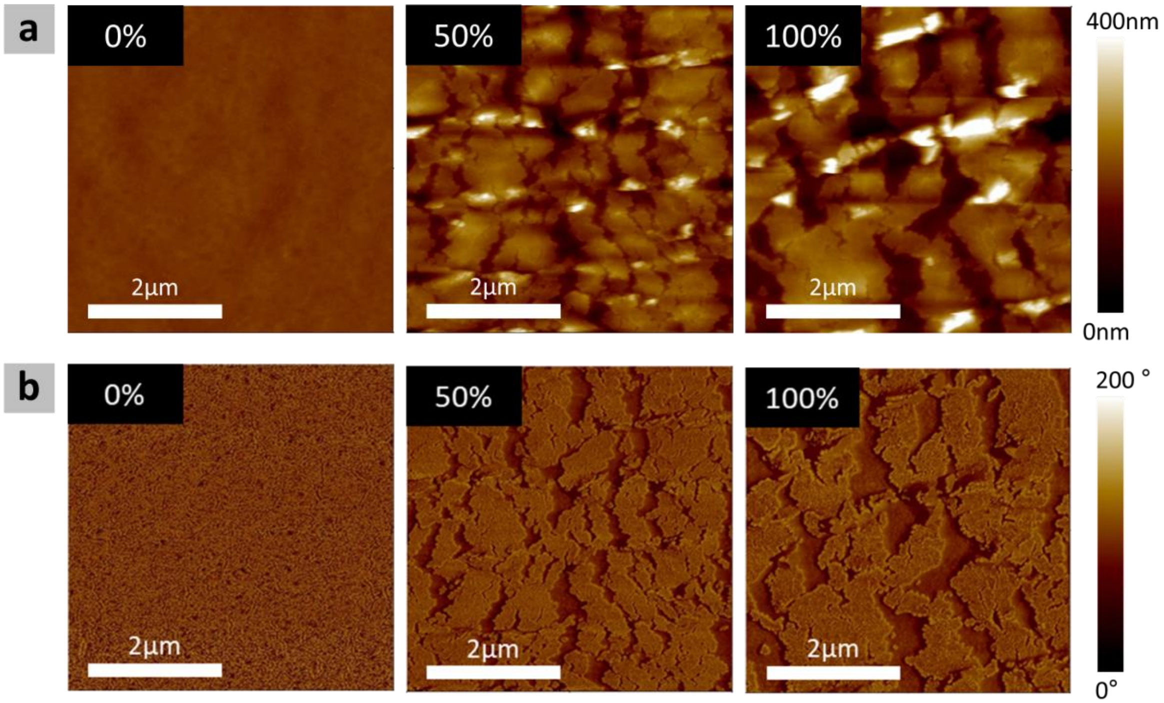

Fig. S30. Morphology analysis of stretchable semiconductor metallization using AFM. (a) Height and (b) phase images of Ag metallized DPPT-TT:SEBS semiconducting film on various strains (0%, 50% and 100% strain).

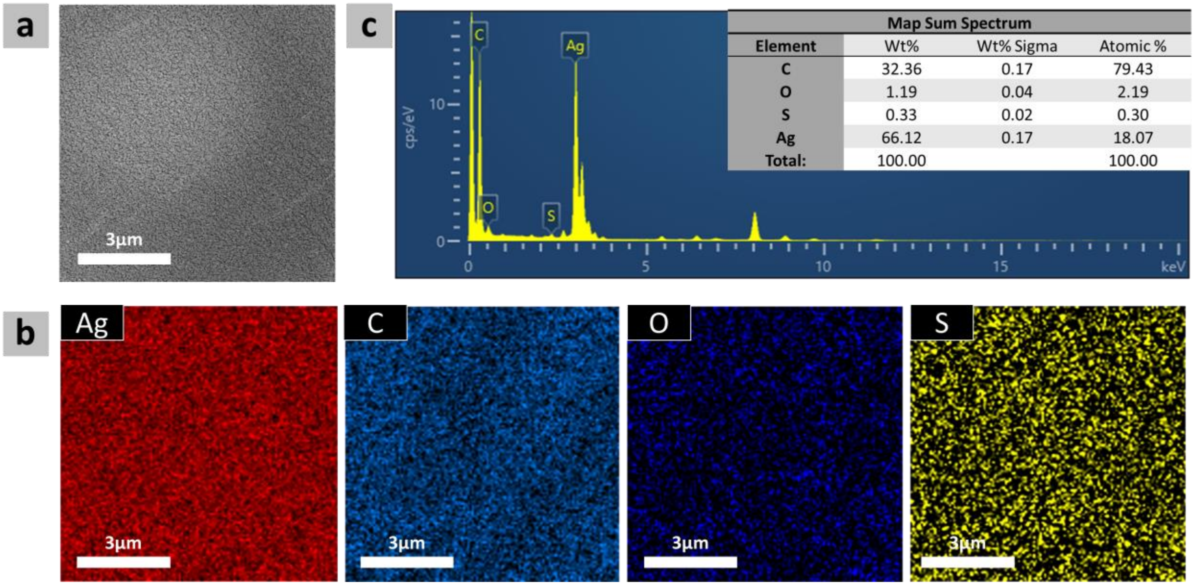

Fig. S31. STEM analysis of pristine Ag metallized DPPT-TT:SEBS film (a) STEM image, (b) its EDS element mapping (Ag, C, O, and S atoms) images and (c) component analysis of EDS mapping images for Ag, O, C and S atom.

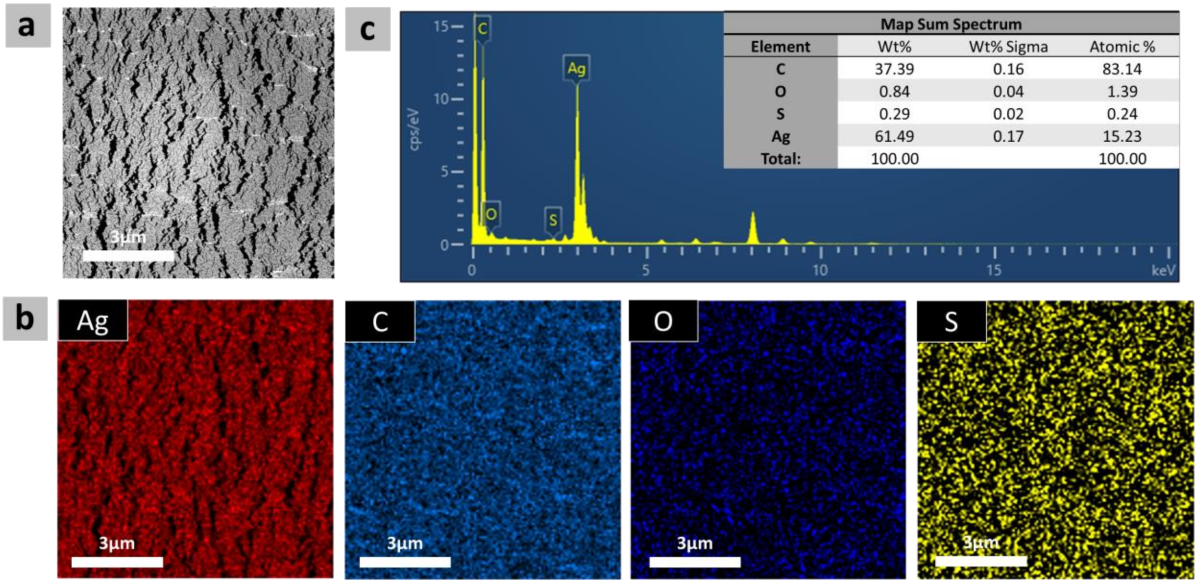

Element	Wt%	Wt% Sigma	Atomic %
C	37.39	0.16	83.14
O	0.84	0.04	1.39
S	0.29	0.02	0.24
Ag	61.49	0.17	15.23
Total:	100.00		100.00

Fig. S32. STEM analysis of Ag metallized DPPT-TT:SEBS film on 50% strain (a) STEM image, (b) its EDS element mapping (Ag, C, O, and S atoms) images and (c) component analysis of EDS mapping images for Ag, O, C and S atom.

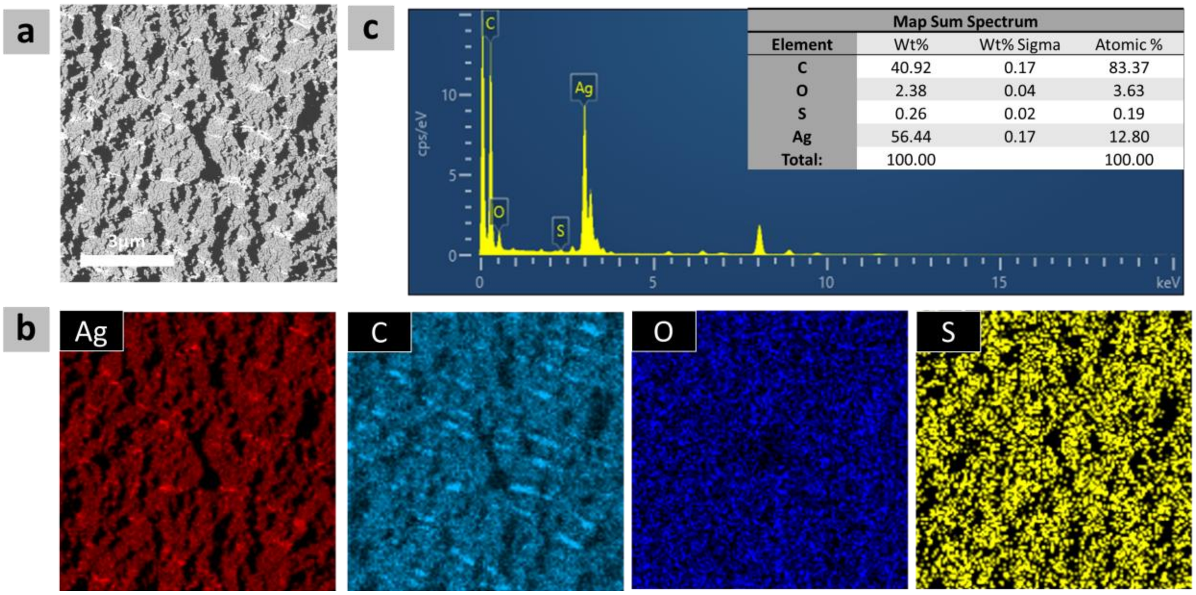

Fig. S33. STEM analysis of Ag metallized DPPT-TT:SEBS film on 100% strain (a) STEM image, (b) its EDS element mapping (Ag, C, O, and S atoms) images and (c) component analysis of EDS mapping images for Ag, O, C and S atom.

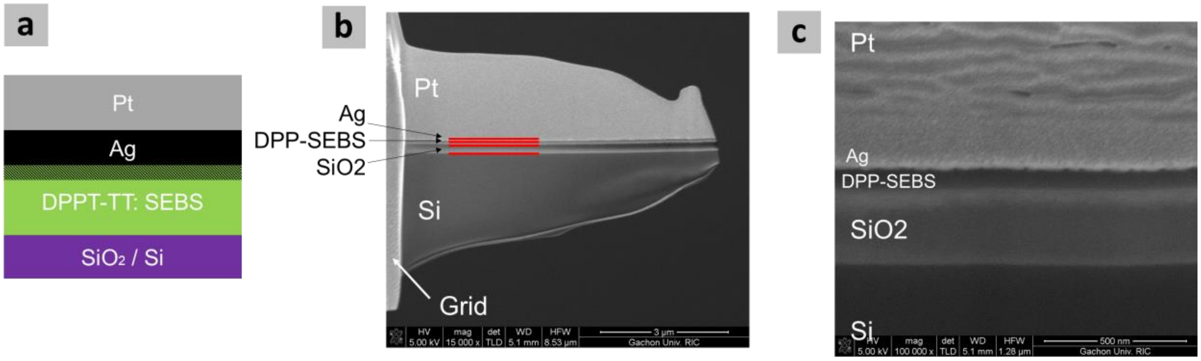

Fig. S34. Cross-section TEM analysis of Ag metallized DPPT-TT:SEBS film. (a) Schematic of sample for analysis. Cross-section TEM images of Ag metallized DPPT-TT:SEBS film through FIB process. (b) micro-and (c) submicron scale magnitude.

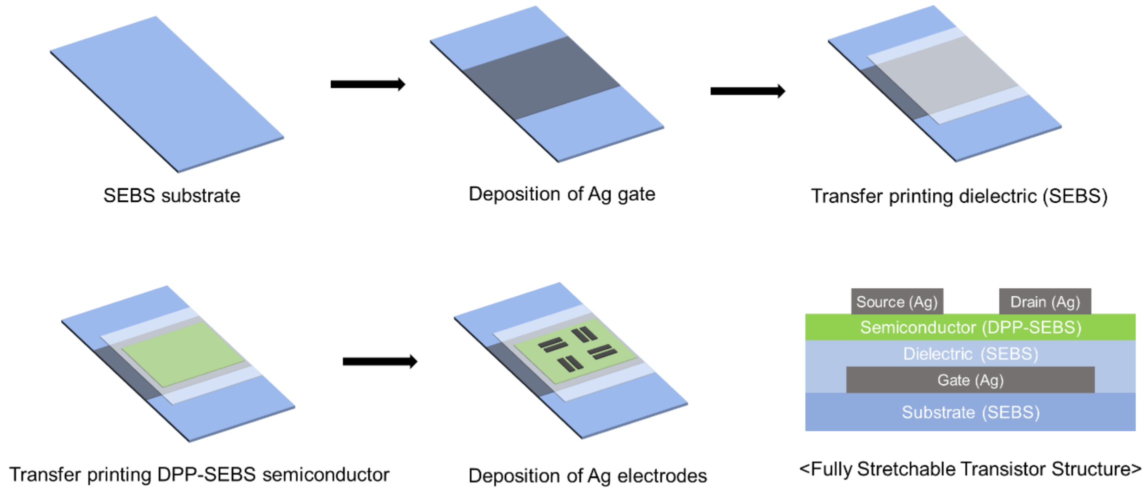

Fig. S35. Fabrication procedure of fully stretchable organic transistor with Ag metalliation.

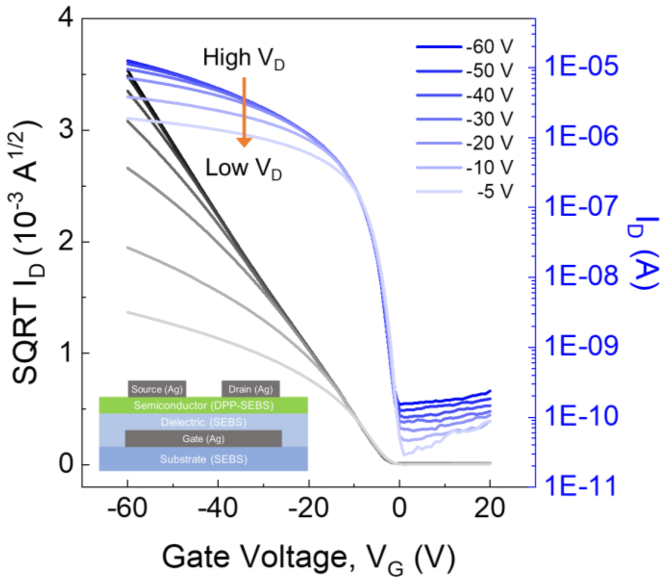

Fig. S36. Transfer curve of OTFT device with Ag electrodes and estimating electrical working property during drain voltage sweep (from 5 V to 60 V)

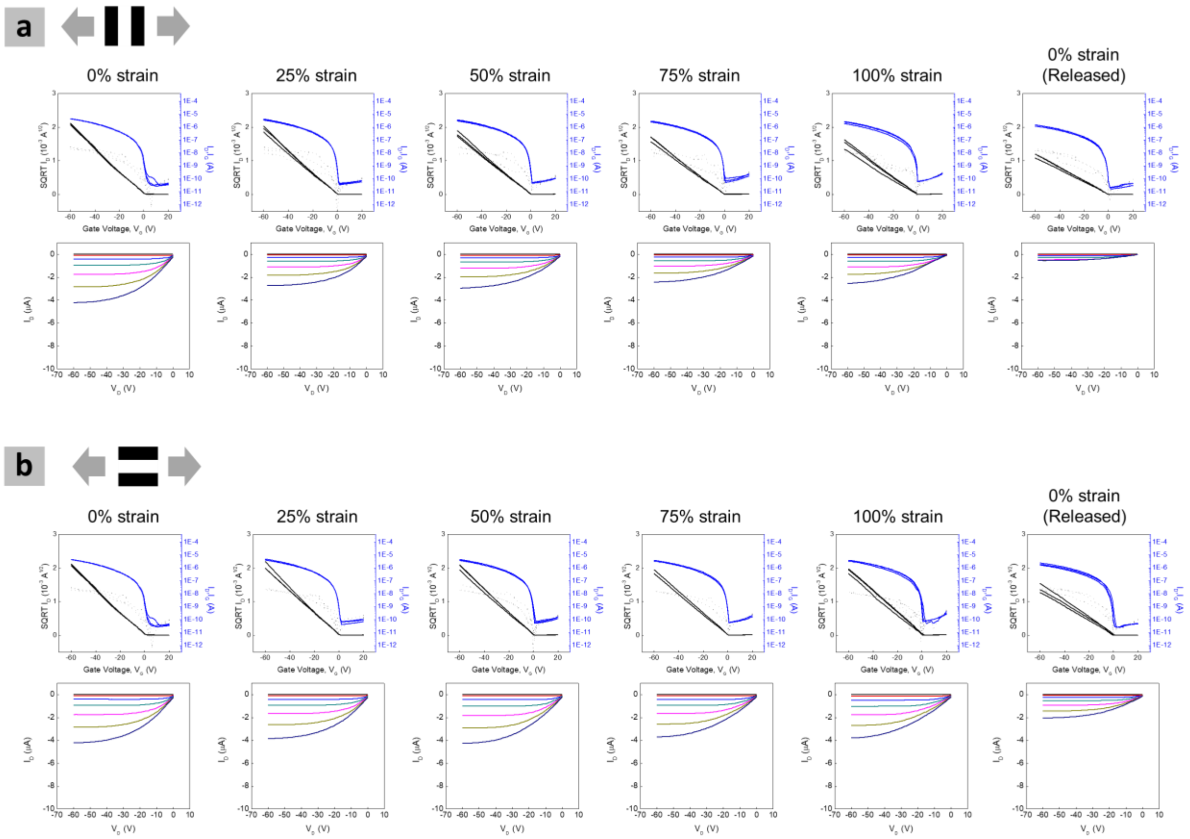

Fig. S37. Transfer and output curves of fully stretchable transistors fabricated with Ag metallization during 100 % stretching cycle according to stretching direction: (a) channel length direction and (b) channel width direction.

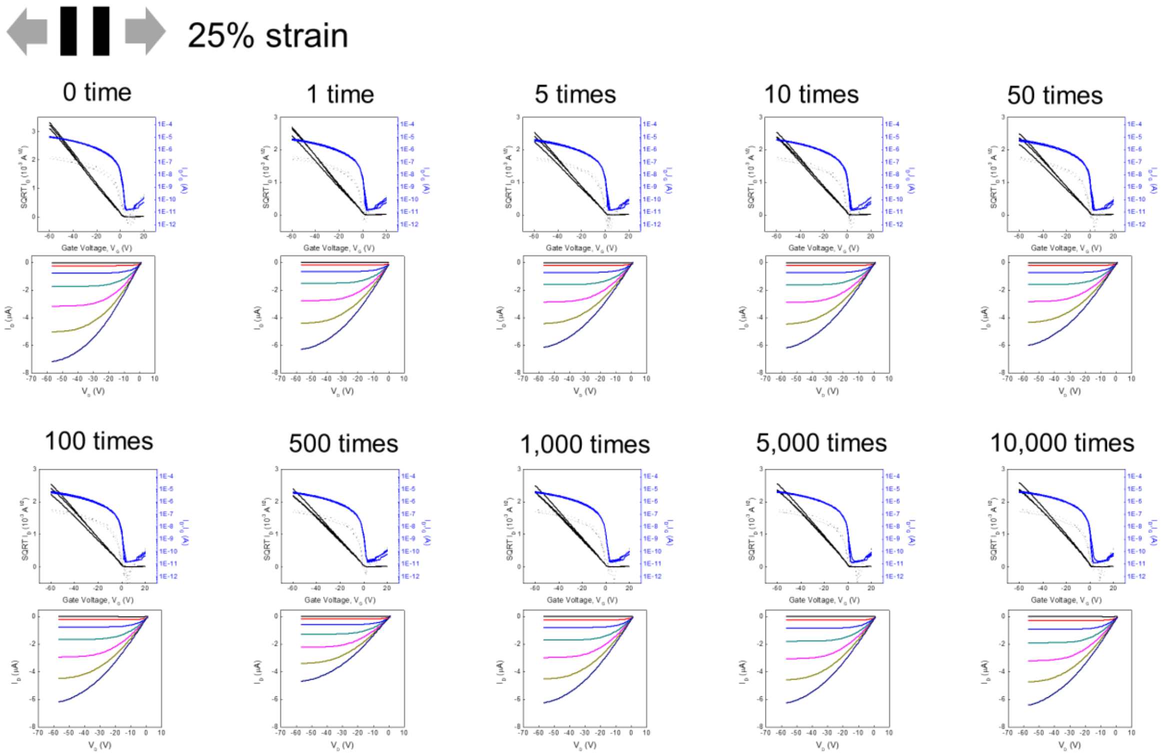

Fig. S38. Transfer and output curves of fully stretchable transistors fabricated with Ag metallization during multiple stretching cycles to channel length direction at 25% strain up to 10,000 times

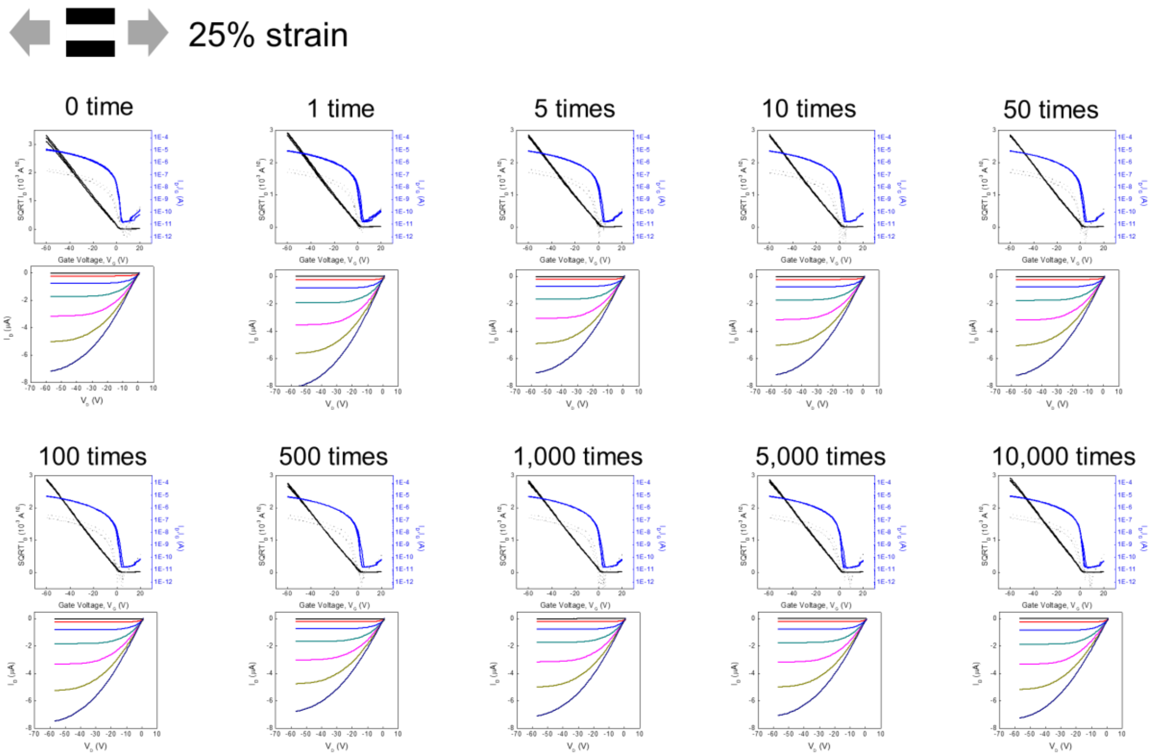

Fig. S39. Transfer and output curves of fully stretchable transistors fabricated with Ag metallization during multiple stretching cycles to channel width direction at 25% strain up to 10,000 times

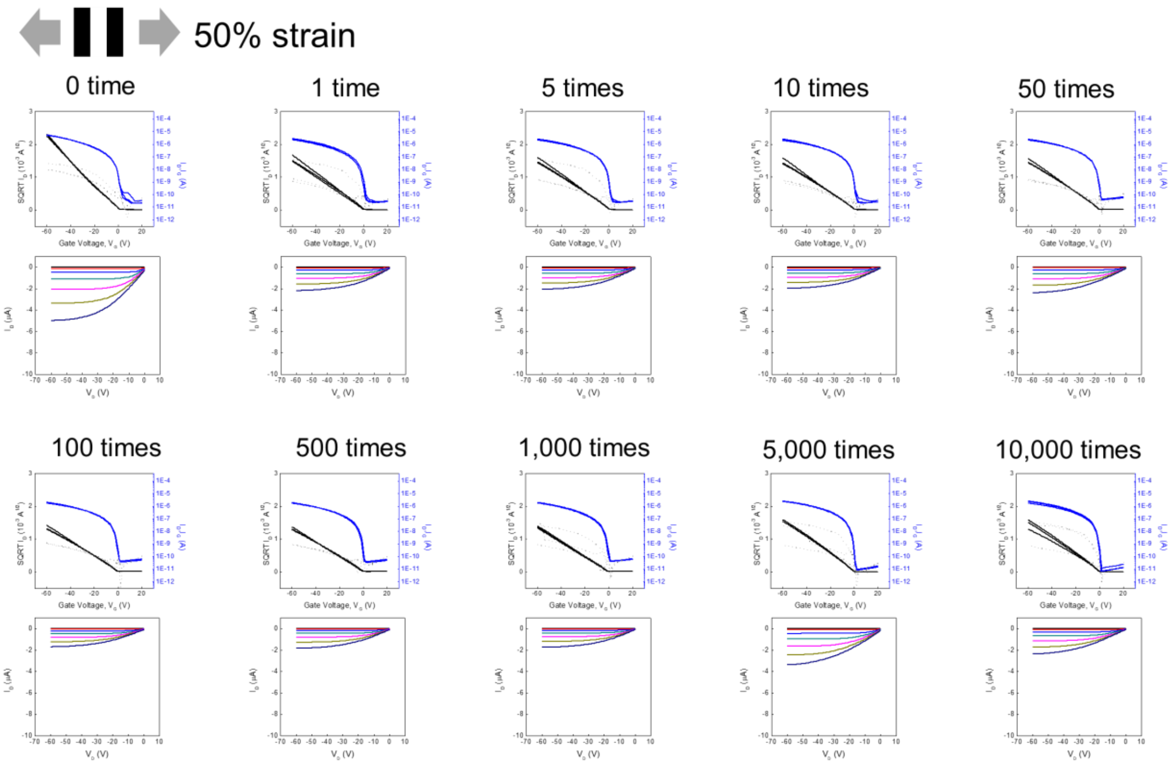

Fig. S40. Transfer and output curves of fully stretchable transistors fabricated with Ag metallization during multiple stretching cycles to channel length direction at 50% strain up to 10,000 times

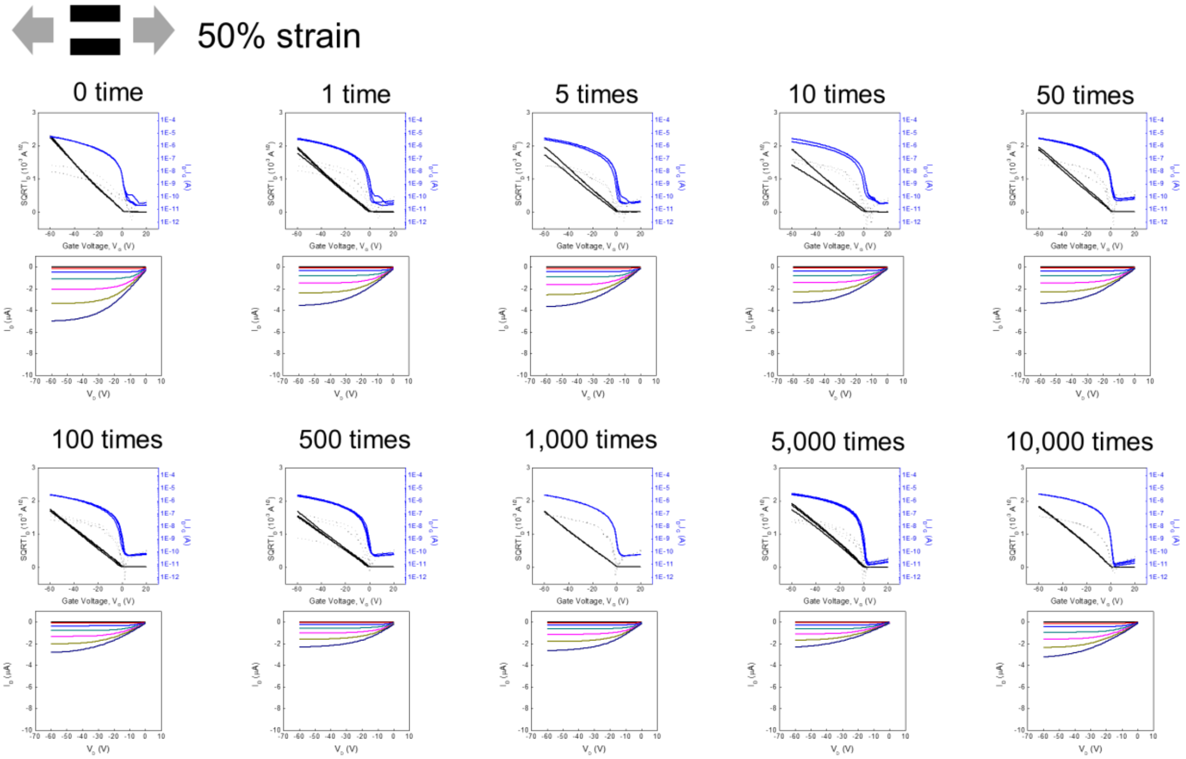

Fig. S41. Transfer and output curves of fully stretchable transistors fabricated with Ag metallization during multiple stretching cycles to channel width direction at 50% strain up to 10,000 times

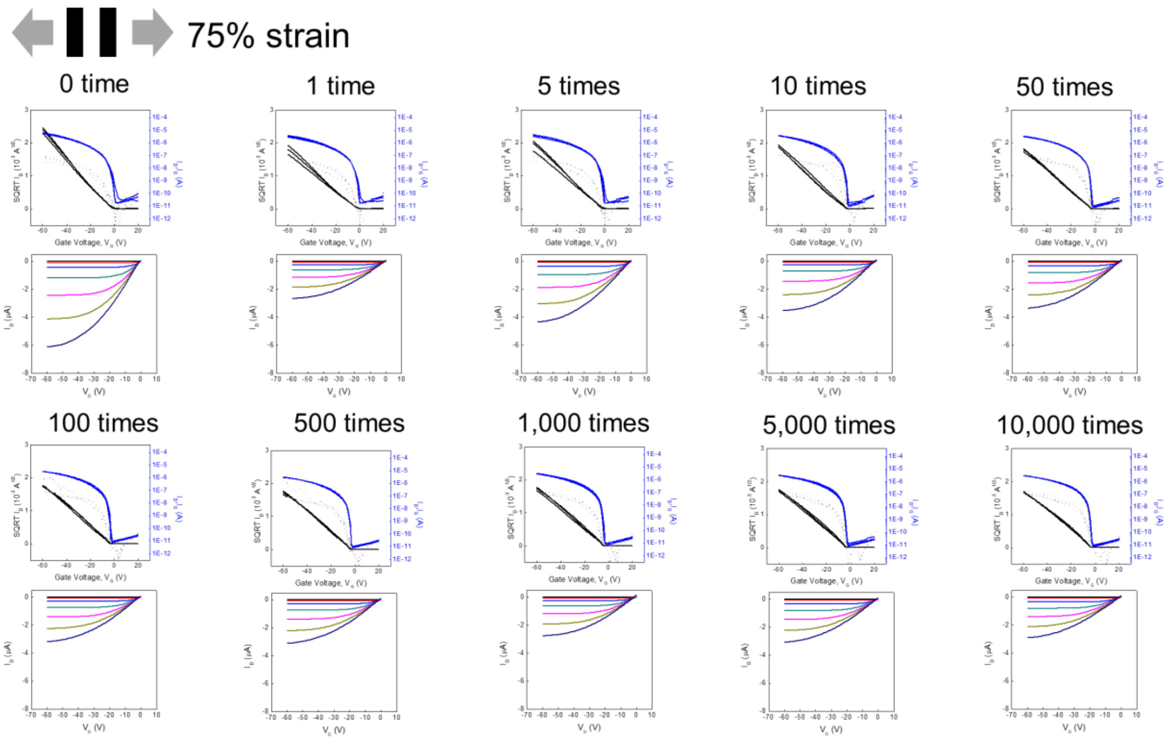

Fig. S42. Transfer and output curves of fully stretchable transistors fabricated with Ag metallization during multiple stretching cycles to channel length direction at 75% strain up to 10,000 times

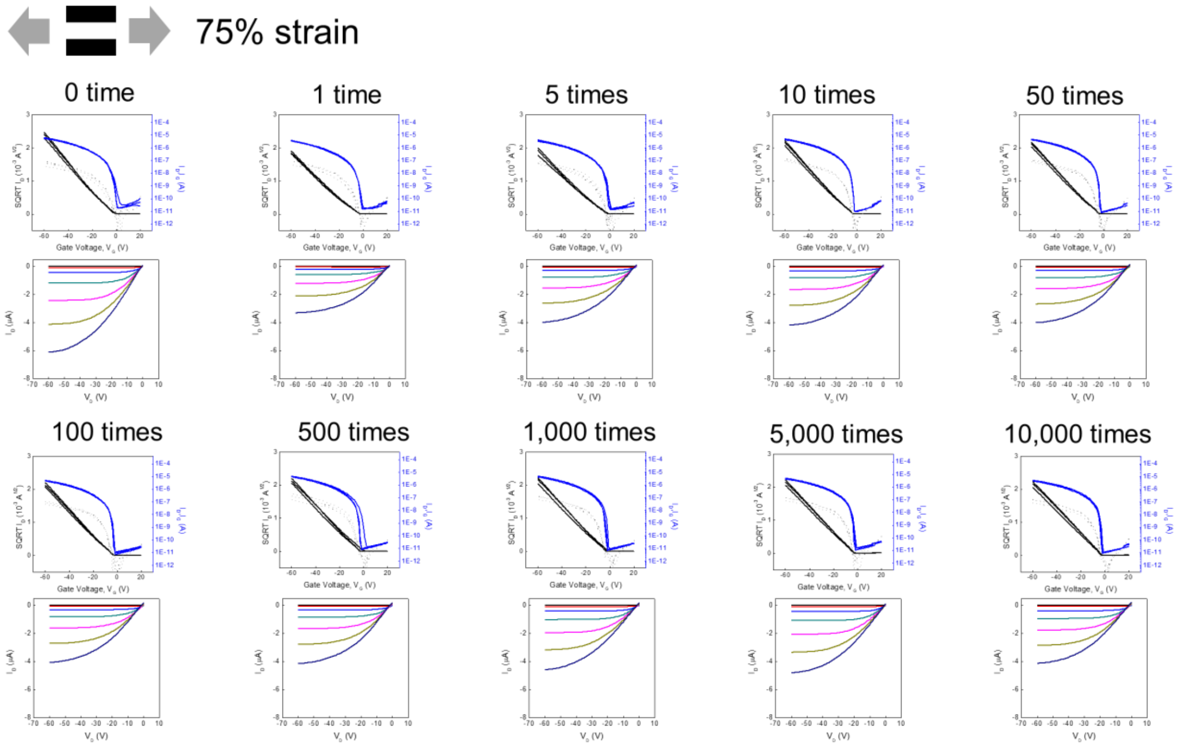

Fig. S43. Transfer and output curves of fully stretchable transistors fabricated with Ag metallization during multiple stretching cycles to channel width direction at 75% strain up to 10,000 times

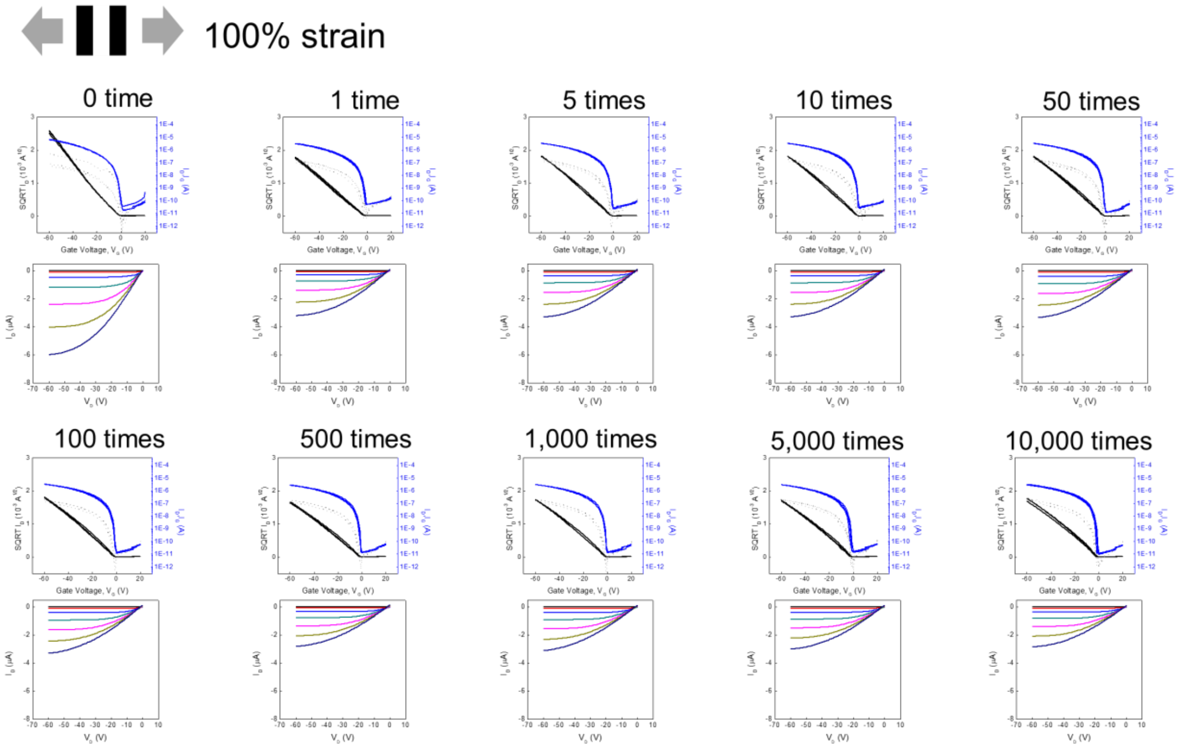

Fig. S44. Transfer and output curves of fully stretchable transistors fabricated with Ag metallization during multiple stretching cycles to channel length direction at 100% strain up to 10,000 times

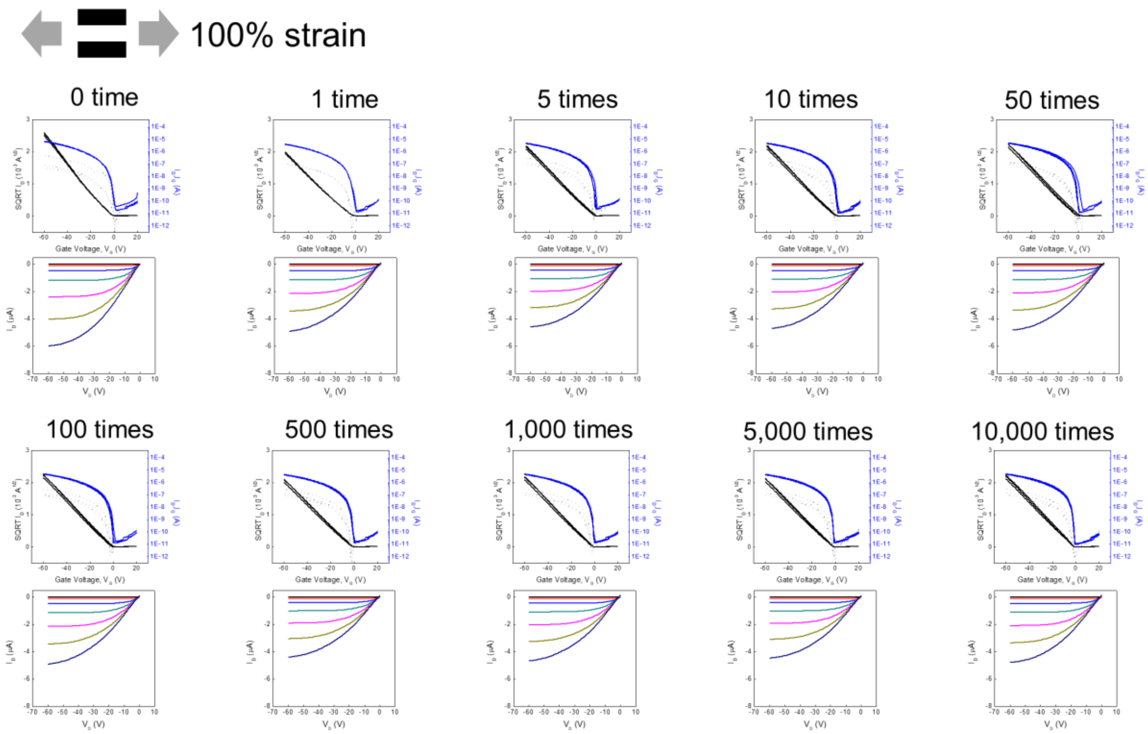

Fig. S45. Transfer and output curves of fully stretchable transistors fabricated with Ag metallization during multiple stretching cycles to channel width direction at 100% strain up to 10,000 times

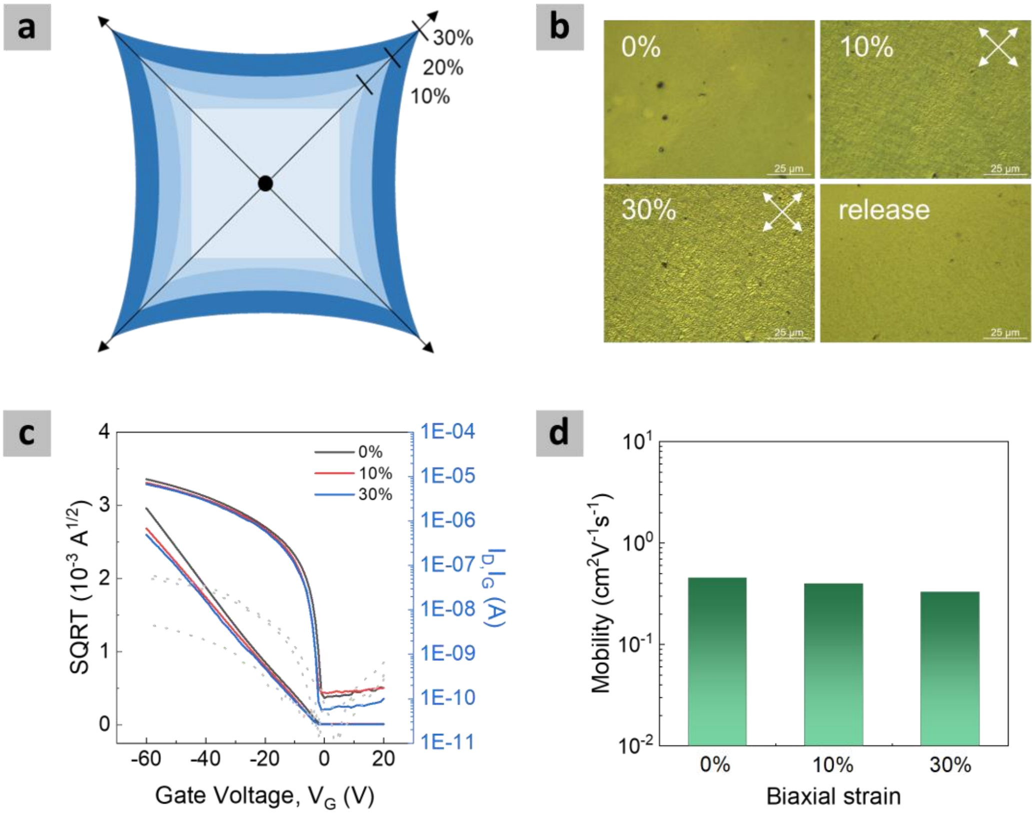

Fig. S46. Biaxial stretching test of stretchable metallized OTFTs. (a) A schematic illustration of biaxial stretching. (b) OM images of Ag metallized film during biaxial stretching. (c) Transfer curves and (d) calculated mobilities of Ag metallized stretchable OTFTs during biaxial stretching (up to 30 % strain).

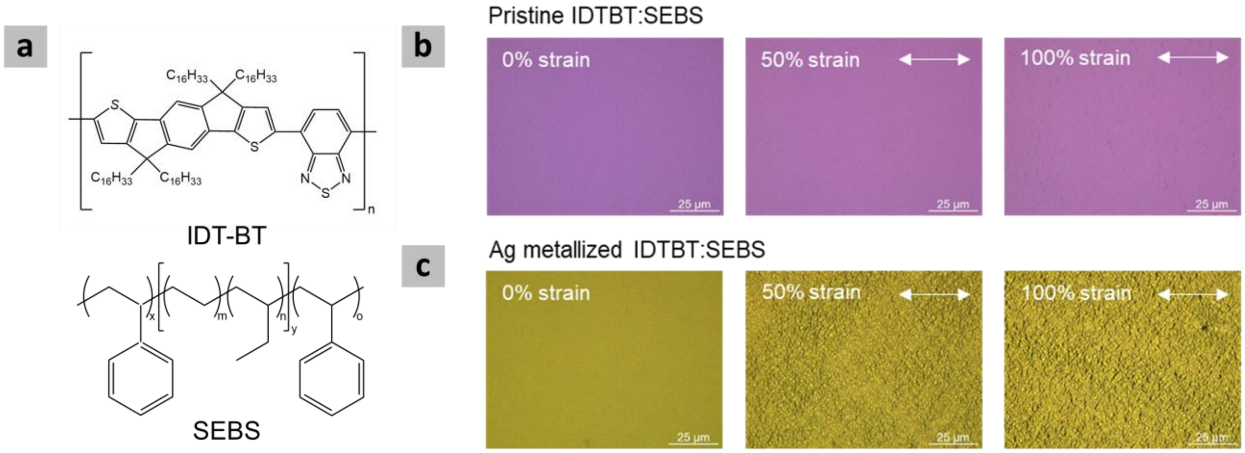

Fig. S47. Expandability test of stretchable metallization for other polymer semiconductor. (a) Molecular structures of IDT-BT and SEBS and optical microscope (OM) images of (b) IDT-BT:SEBS films and (c) Ag metallized (50nm) IDT-BT:SEBS films as a function of strain.

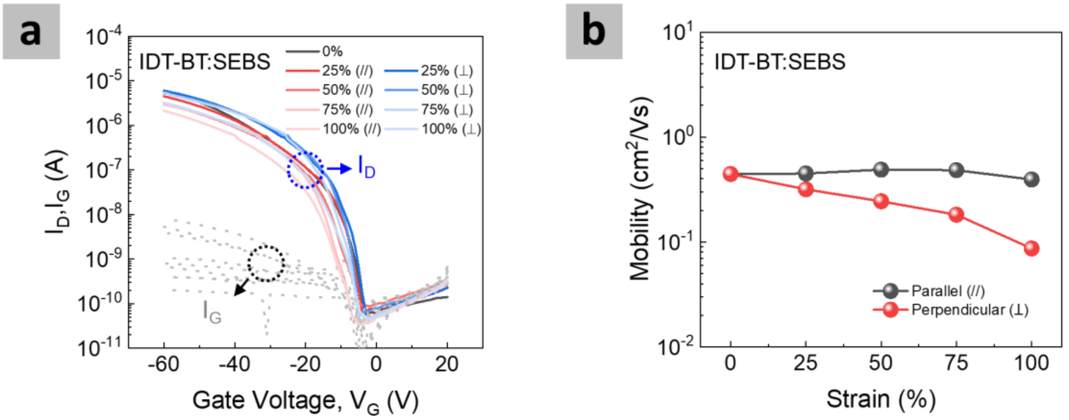

Fig. S48. Expandability test of stretchable metallization for other polymer semiconductor. (a) Transfer curves and (b) field-effect mobilities of Ag metallized IDT-BT:SEBS based stretchable OFETs as a function of strain.

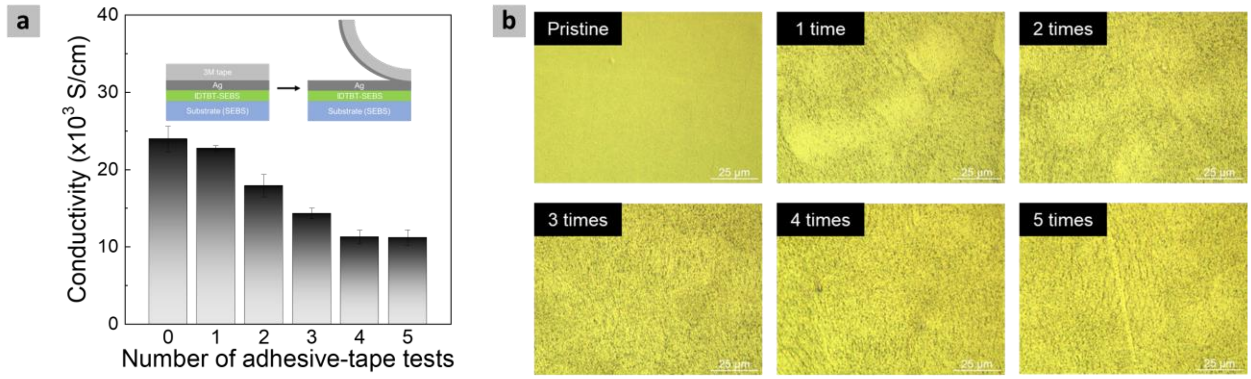

Fig. S49. Expandability test of stretchable metallization for other polymer semiconductor. (a) Conductivity of Ag metallized IDT-BT:SEBS film and (b) optical microscope (OM) images of Ag metallized IDT-BT:SEBS film during multiple peel-off tests using 3M tape (adhesion force: 2.2 N/cm)

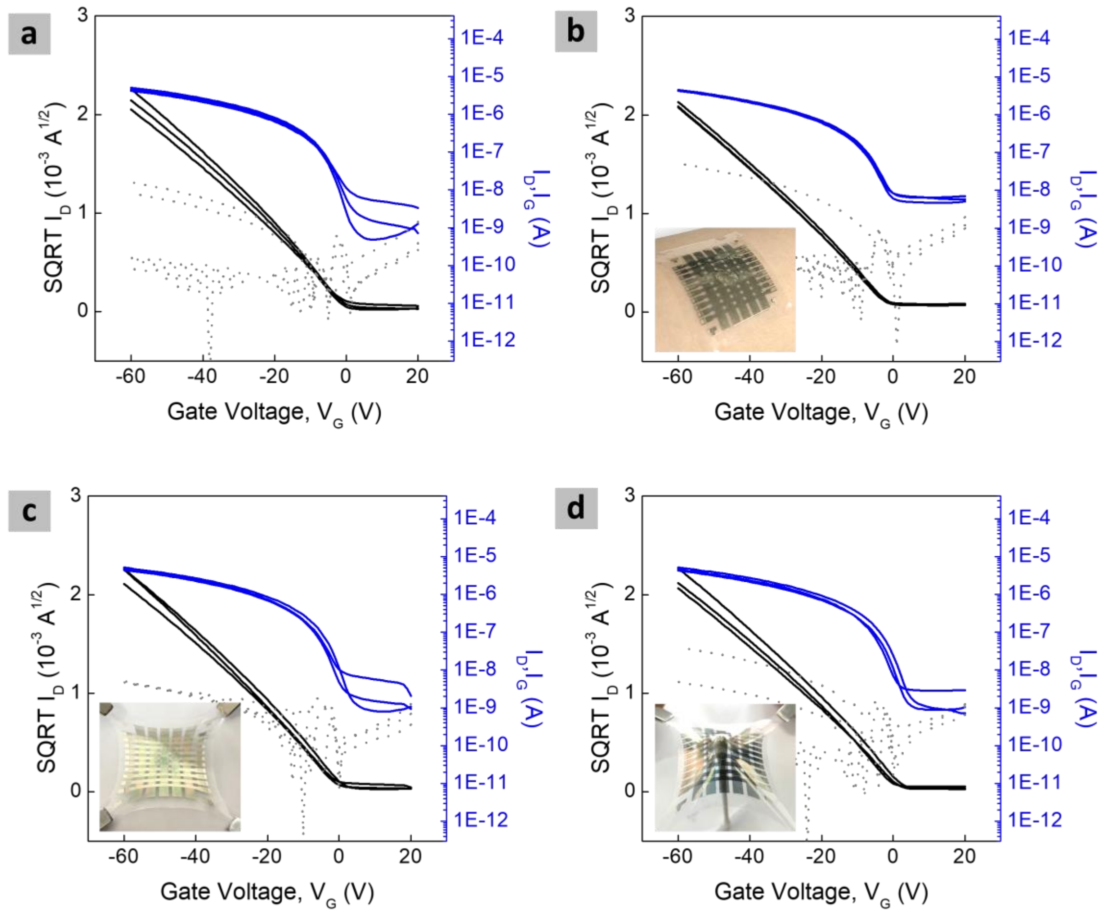

Fig. S50. Transfer curve characteristics of fully stretchable organic transistor active-matrix array fabricated with Ag metallization (a) on probe station, (b) on human skin, (c) under biaxial stretching (30 %) and (d) under indenting (depth: 1cm) with cotton swap.

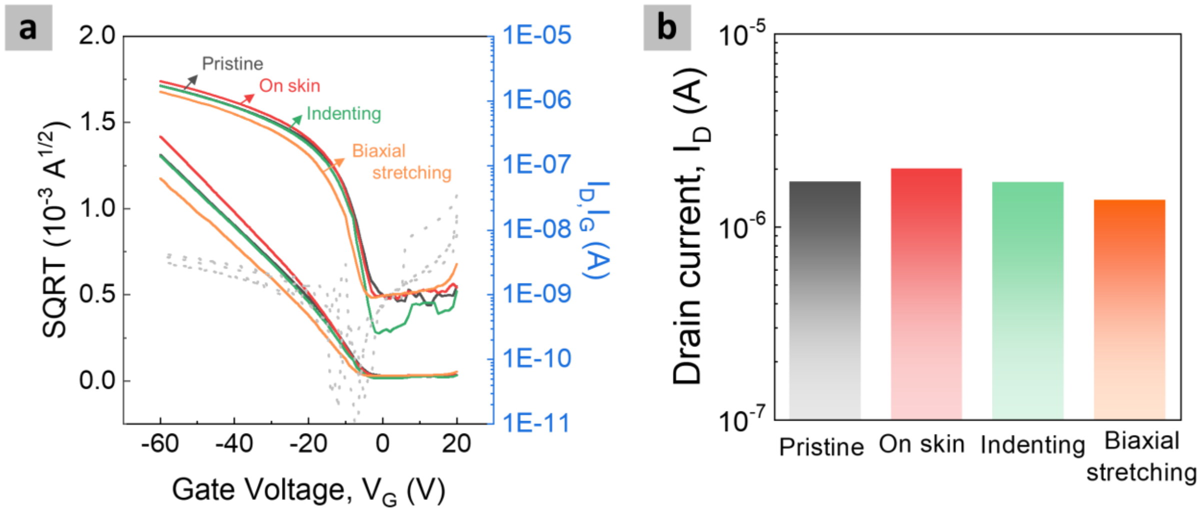

Fig. S51. In-situ measured transfer curve characteristics of fully stretchable organic transistor active-matrix array in Movie S2 (a) under various deformations (pristine, on human skin, indenting with cotton swap and biaxial stretching, and (b) theses on currents at 60 V (drain and gate) in series.

Table S1. Performance comparison of stretchable OTFTs and the specific information.

Device Structure	Semiconductor	Initial OFET Performance	Stretching Durability	Electrode Material (method)	Electrode conductivity (σ) or resistance (R)	Electrode resistance on strain	Electrode Peel-Off Test	Ref.
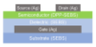	**DPPT-TT:SEBS**	**μ : 0.294 $cm^2V^{-1}s^{-1}$<br>On/Off : ~10^4**	**μ : 0.191 $cm^2V^{-1}s^{-1}$<br>On/Off : ~10^4<br>Strain : 100%<br>Cycles : 10,000**	**Ag metallization<br>(thermal evaporation)**	σ : 12532.2 S/cm<br>R : 6.54 Ω	R : 9.36 Ω<br>under 100% stain	**" Conductivity "<br>Pristine: 12,956 S/cm<br>After test: 3,460 S/cm,<br>Test Number: 5<br>Adhesion force : 2.2 N/cm<br>Peeling rate: 2.4 cm/s<br>(3M Scotch® Magic™ Tape 810)**	**This work**
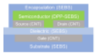	DPPT-TT:SEBS	μ : 0.59 $cm^2V^{-1}s^{-1}$<br>On/Off : ~10^5	μ : 0.24 $cm^2V^{-1}s^{-1}$<br>On/Off : ~10^5<br>Strain : 25%<br>Cycles : 1,000	CNT<br>(spray coating)	unmentioned	unmentioned	N/A	Ref. 4
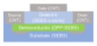	DPP-SVS:SEBS	μ : 0.821 $cm^2V^{-1}s^{-1}$<br>On/Off : ~10^4	μ : N/A<br>On/Off : ~10^3<br>Strain : 100%<br>Cycles : 1,000	CNT<br>(spray coating)	R : 1.1 MΩ	R : 4.5 MΩ<br>under 100% strain	N/A	Ref. 12
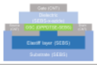	DPPDTSE:SEBS	μ : 0.62 $cm^2V^{-1}s^{-1}$<br>On/Off : ~10^4	μ : N/A<br>On/Off : N/A<br>Strain : 100%<br>Cycles : 1,000	CNT<br>(spray coating)	R : 2000 Ω/□	R : 18,000 Ω/□<br>under 100% strain	N/A	Ref. 5
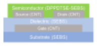	DPPDTSE:SEBS	μ : 0.40 $cm^2V^{-1}s^{-1}$<br>On/Off : ~10^4	μ : N/A<br>On/Off : ~10^4<br>Strain : 50%<br>Cycles : 1,000	CNT<br>(spray coating)	unmentioned	unmentioned	N/A	Ref. 11
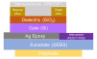	SWCNT	μ : 1.24 $cm^2V^{-1}s^{-1}$<br>On/Off : ~10^5	N/A	PEDOT:PSS<br>(inkjet printing)	σ : 1000 S/cm	unmentioned	N/A	Ref. 24
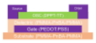	DPPT-TT	μ : 0.255 $cm^2V^{-1}s^{-1}$<br>On/Off : ~10^4	μ : N/A<br>On/Off : N/A<br>Strain : 50%<br>Cycles : 1,000	PEDOT:PSS<br>(spray coating)	σ : 525 S/cm	unmentioned	N/A	Ref. 23
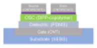	DPPT-TVT-PDCA	μ : 0.342 $cm^2V^{-1}s^{-1}$<br>On/Off : ~10^5	μ : 0.165 $cm^2V^{-1}s^{-1}$<br>On/Off : ~10^5<br>Strain : 25%<br>Cycles : 500	PEDOT:PSS/CNT<br>(spray coating)	unmentioned	unmentioned	N/A	Ref. 3
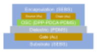	DPP-PDCA-PDMS	μ : 0.11 $cm^2V^{-1}s^{-1}$<br>On/Off : ~10^5	N/A	Au<br>(thermal evaporation)	R : 36 Ω	R : 140 Ω<br>under 100% stain	N/A	Ref. 8
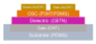	P3HT:PDMS	μ : 0.48 $cm^2V^{-1}s^{-1}$<br>On/Off : ~10^4	N/A	Au/CNT<br>(thermal evaporation/spray coating)	R : 11 Ω	R : 26 Ω<br>under 50% stain	N/A	Ref. 7
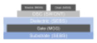	SWCNT	μ : 5.6 $cm^2V^{-1}s^{-1}$<br>On/Off : ~10^3	N/A	Graphene<br>(CVD)	unmentioned	unmentioned	N/A	Ref. 15

Table S2. Capacitance change of SEBS dielectric according to stretching direction.

Stretching direction	Strain (%)	Channel length (μm)	Channel width (μm)	Capacitance (nF/cm^2)
Parallel to channel length (//)	0	150	975	1.416
	25	177	912.2	1.443
	50	213.8	864	1.455
	75	228.6	818	1.521
	100	276.2	747	1.76
	0 (released)	156.8	960.4	1.498
Perpendicular to channel width (⊥)	0	150	1022	1.392
	25	141.2	1230	1.628
	50	132	1394.2	1.753
	75	124.2	1569	1.791
	100	114.2	1939	1.87
	0 (released)	149.2	1088.8	1.462

Table S3. Device geometry and capacitance changes of SEBS dielectric on biaxial stretching.

Biaxial strain (%)	Channel length (μm)	Channel width (μm)	Capacitance (nF/cm^2)
0	153.41	981	1.784
10	176.84	1032.2	1.894
30	199.96	1148.1	2.209
